# Evidence of populational *Burkholderia pseudomallei* exposure in Madagascar

**DOI:** 10.1371/journal.pntd.0013419

**Published:** 2025-11-18

**Authors:** Solohery Lalaina Razafimahatratra, Sarobidy Tsiory Avotra Andrianambinintsoa, Lova Tsikiniaina Rasoloharimanana, Minoarisoa Rajerison, Voahangy Rasolofo, Carina M. Hall, Kimberly R. Celona, Jinhee Yi, Paul Keim, David M. Wagner, Erik W. Settles, Matthieu Schoenhals

**Affiliations:** 1 Immunology of Infectious Diseases, Institut Pasteur de Madagascar, Antananarivo, Madagascar; 2 Plague Unit, Institut Pasteur de Madagascar, Antananarivo, Madagascar; 3 Scientific Direction, Institut Pasteur de Madagascar, Antananarivo, Madagascar; 4 The Pathogen and Microbiome Institute, Northern Arizona University, Flagstaff, Arizona, United States of America; 5 Department of Biological Sciences, Northern Arizona University, Flagstaff, Arizona, United States of America; 6 Immunology Department, Institut Pasteur, Paris, France; University of Connecticut College of Agriculture Health and Natural Resources, UNITED STATES OF AMERICA

## Abstract

**Background:**

*Burkholderia pseudomallei* (Bp), the causative agent of melioidosis, poses a significant health risk in endemic regions. This study aimed to characterize six previously identified Bp antigens for exposure monitoring in potential endemic setting of Madagascar and to assess potential exposure and reactivity across Madagascar to inform diagnostic strategies and understand regional exposure patterns.

**Methods:**

Six highly reactive Bp antigens, CPS I, LPS types A and B, HCP1, AhpC, and GroEL, were characterized using the protein BLAST (BLASTp) algorithm against the NCBI non-redundant protein database to evaluate conservation and specificity. Serological reactivity was analyzed in 5,736 serum samples from six regions of Madagascar using Luminex bead-based multiplex assays. Principal Component Analysis was conducted to identify co-reactivity patterns. Environmental sampling in Mahajanga assessed the presence of Bp DNA in soil and water samples via real-time PCR.

**Results:**

Antigen conservation varied, with HCP1 and CPS I demonstrating the highest specificity, suggesting their potential for targeted serological detection. Seroprevalence and co-seroprevalence were highest in the Mahajanga region, particularly for HCP1 and CPS I (p < 0.0001). PCA revealed distinct antigen-specific immune response profiles. Environmental sampling confirmed the presence of Bp DNA in a water sample from Mahajanga, indicating local bacterial presence in the environment.

**Conclusions:**

These findings suggest human exposure to Bp occurs in multiple regions in Madagascar. HCP1 and CPS I emerged as promising targets for diagnostic applications, and environmental detection of Bp underscores the need for targeted public health interventions in higher-risk regions like Mahajanga.

## Author summary

Melioidosis is a serious and potentially fatal infectious disease caused by the environmental bacterium *Burkholderia pseudomallei* (Bp), which is naturally found in soil and surface water. The disease primarily affects individuals with underlying conditions such as diabetes and can lead to severe illness or death if not promptly diagnosed and treated. Despite its clinical severity, melioidosis often goes undiagnosed in low-resource settings due to the need for specialized laboratory diagnostics.

In Madagascar, only a handful of human cases have been officially reported, but the true extent of exposure across the population has remained unknown. To address this gap, we conducted a large-scale serological survey, analyzing blood samples from more than 5,000 healthy blood donors across five regions of the country. By screening for antibodies indicative of past infection, we uncovered a significantly higher seroprevalence in the coastal region of Mahajanga, pointing to localized transmission. Supporting these findings, we also detected *B. pseudomallei* DNA in surface water samples from the same region, providing environmental evidence of exposure risk.

These results suggest that melioidosis may be more widespread in Madagascar than previously recognized. Our study underscores the importance of strengthening disease surveillance and environmental monitoring while raising awareness of this neglected tropical disease. It also highlights the potential of using blood donor samples as a cost-effective and scalable tool for uncovering hidden infectious threats in resource-limited settings.

## Introduction

*Burkholderia pseudomallei* (Bp), an environmental Gram-negative bacterium, is the causative agent of melioidosis in humans and animals [1]. Infection occurs through environmental exposure mainly via percutaneous inoculation, inhalation, or ingestion [2,3]. Although the majority of cases are sporadic, as human-to-human transmission is exceedingly rare, small clusters of human cases have been associated with contaminated products, water supplies, or environments [4–6]. Most individuals exposed to Bp do not develop melioidosis; however, diabetes and other conditions that impair innate and adaptive immune responses significantly increase the risk of disease [7,8].

Recent modelling estimates suggest that there may be up to 165,000 human melioidosis cases annually worldwide, with an estimated 89,000 resulting in death [9]. Reported mortality rates range from under 10% to over 40% [7,8]. Most reported cases occur in Southeast Asia and northern Australia, but Bp is increasingly being reported from many other regions of the world, especially the Pacific, South Asia, Africa, and the Americas [10–15]. The western Indian Ocean islands have reported several cases: two individuals were diagnosed in Mauritius in 2004 and 2006 [16]. Seven cases were reported in the Réunion Island [17]. Additionally, two cases were reported in Seychelles in 2013 [18].

In Madagascar, Bp was first isolated in Antananarivo in 1932 in a guinea pig inoculated with a submaxillary node of a slaughtered pig [19]. The presence of this bacterium was further confirmed in 1977 by Galimand and Dodin who isolated and identified the bacterium from soil samples collected from the Antananarivo Zoo and a pig farm in the same city [20]. Since 2004, six human cases of melioidosis have been identified, all originating from Mahajanga on Madagascar’s west coast. Three cases were diagnosed and treated in Réunion Island in 2004, 2005, and 2016. Two autochthonous cases were diagnosed in 2013 and Bp isolated in the yard of one of them in 2014, and one traveller was diagnosed after returning to Belgium in 2018 [21]. Taken together, these sporadic cases suggest that robust environmental and clinical surveillance systems may need to be implemented, particularly in potentially high-risk areas like Mahajanga.

The current gold standard for melioidosis detection in humans is the culture of Bp from clinical specimens, which offers high specificity (100%) but low sensitivity (~60%) [22]. Culturing also requires specialized laboratory infrastructure, such as a biosafety level 3 laboratory. Serological assays can complement culture diagnosis, with the indirect hemagglutination assay (IHA) being the standard test. The IHA detects IgM antibodies in blood to Bp whole cell lysates (WCL). Although highly specific in non-endemic areas, the IHA faces challenges in endemic regions due to background or cross-reactive seropositivity, maintaining a ~ 56% sensitivity [23,24]. New serological diagnostic tools aim to improve upon the IHA for timely and effective diagnosis of melioidosis, though none are currently approved for routine use [25–35].

Multiplex assays incorporating various Bp immunogenic antigens have enhanced the power of serological detection. A 2D microarray detection assay using 20 Bp proteins surpassed the IHA in sensitivity and specificity (86.7% vs. 57%, and 97% vs. 96%) [29]. Highly immunogenic antigens like O-polysaccharide (OPS), Capsular polysaccharide (CPS), and Hemolysin co-regulated protein 1 (HCP1) have also been incorporated into rapid enzyme-linked immunosorbent assays (ELISA) [26]. A novel serodiagnostic bead assay, known as BurkPx that capitalized upon host response to multiple antigens, has been developed to enhance the accuracy of melioidosis diagnosis. This assay demonstrates a sensitivity of 90% and a specificity of 93%, outperforming any single antigen in direct comparisons. This improvement in diagnostic accuracy surpasses that of currently available serological assays, offering a more reliable tool for the detection of melioidosis [36].

Surveillance of Bp presents significant challenges, especially in resource-limited settings like Madagascar. But the establishment of a serum biobank from five Regional Blood Transfusion Centers (RBTC) in Madagascar since 2020 provides a valuable resource for enhancing screening and surveillance efforts [37–39]. Indeed, it has been demonstrated that blood donors can serve as a representative sample of the general population by applying direct standardization and Bayesian modelling to adjust for demographic and geographic differences. Using data stratified by gender, age, and region, and referencing the 2018 Malagasy Population and Housing Census, we found that estimates corrected using population weights or Bayesian models closely matched crude values [37]. Although blood donors may slightly overrepresent urban populations compared to those from rural areas, their demographic similarity to the broader population and the consistency of adjusted and crude estimates highlights the utility of blood donors as a sentinel population for monitoring health trends at the regional and national levels in Madagascar [37,38]. The biobank, representative of the general population, comprises serum samples collected over time from diverse groups, facilitating retrospective serological analyses and monitoring antibody responses to Bp. The aim of this study was to utilize serological assays, such as the novel BurkPx bead assay, to identify regional Bp exposure patterns in Madagascar. This approach offered a cost-effective and scalable alternative to direct pathogen detection methods, facilitating broader monitoring of melioidosis across the country follow by focused Bp detection in regions with increase seropositivity.

## Materials and methods

### Sample collection

**Fig 1:**
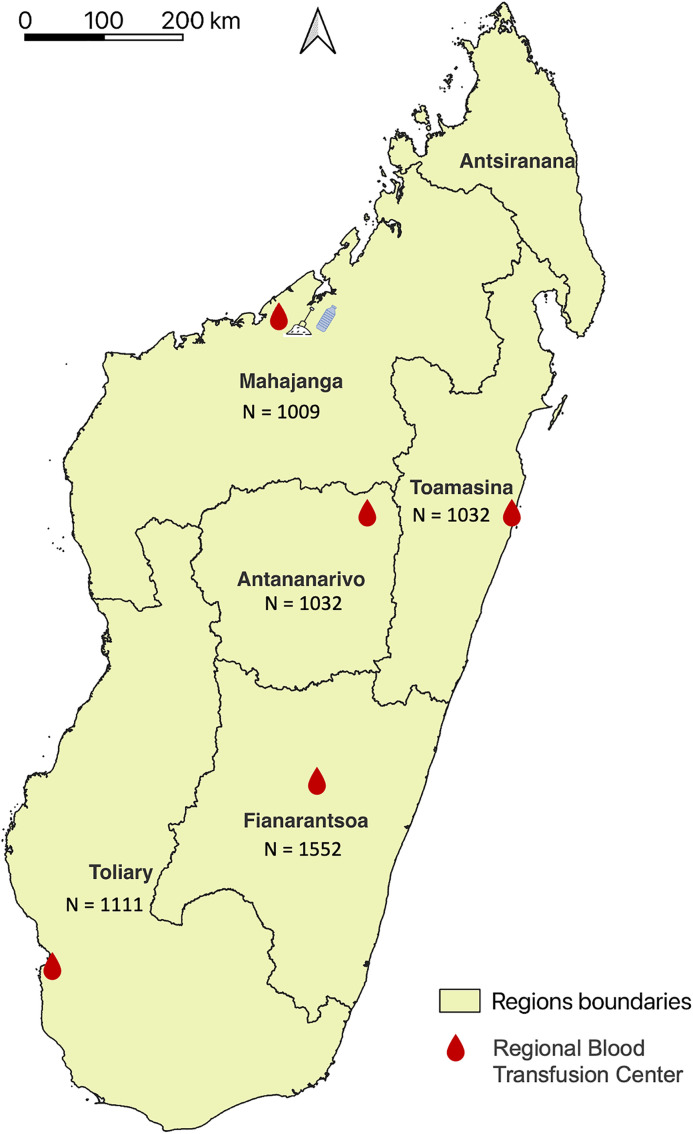
Map of Madagascar indicating the five regional blood transfusion centers from which sera were collected. N, number of collected sera; (*) Reported melioidosis cases from 2004 to 2016. Soil and water sampling locations represented by spade and bottle icons, respectively. Map created in QGIS using base layers from OpenStreetMap. Base map source: OpenStreetMap (public domain). Downloaded from [https://download.geofabrik.de/africa/madagascar.html]. Terms of use: [https://www.openstreetmap.org/copyright].

The study protocol was reviewed and approved by the Comité d’Éthique de la Recherche Biomédicale de Madagascar (CERBM: IORG00001212). The study used anonymized residual serum samples collected during routine blood qualification procedures at five regional blood transfusion centers across the five regions of Madagascar. As these samples were obtained as part of standard screening activities, no formal written consent was required for their secondary use. However, donors were informed of the potential use of their residual samples for public health and research purposes and retained the right to refuse such use.The samples utilized in this study were collected from blood donors between 2020 and 2022 [38,37]. A total of 5,376 serum samples were selected from five regions of Madagascar: Mahajanga (West coast), Toamasina (East coast), Antananarivo (Central highland), Fianarantsoa (Southern highland), and Toliara (South-west), with approximately 1,000 samples collected per region. This sample size provides good statistical power and precision: for each region, the standard error of the prevalence estimate is $\sqrt{\left(P(\text{1}-P)/\text{1}\text{0}\text{0}\text{0}\right)}$, resulting in narrow 95% confidence intervals even for low prevalences. Hence, the chosen sample size is suitable for estimating regional seroprevalence with high precision and for detecting moderate interregional differences, although differences smaller than about 0.5 percentage points among very low-prevalence regions may not be reliably detected. Participants were predominantly males (3,824/4,713 – 81.1%), and aged from 18 to 70 years (Table 1 and Fig 1). In Madagascar, blood donors are primarily family members of hospitalized patients, representing approximately 80% of all blood donors nationally. The condition for receiving one unit of blood is typically the replacement of two units by relatives or acquaintances of the patient. Eligibility criteria for blood donation include being over 18 years of age, in a healthy state at the time of donation, having a minimum weight of 50 kg, and observing a minimum interval between donations—three months for males and four months for females. Women who are pregnant, breastfeeding, menstruating, or using injectable or implant contraceptives are excluded from donation [40].

**Table 1:** Characteristics of participant (n = 5,736).

Variable	n (%)
**Sex**	
Male	4,118 (71.8)
Female	930 (16.2)
N/a	688 (12.0)
Mean age (Min-Max)	32 (18-70)
**Profession**	
Farmer	754 (13.1)
Non Farmer	3,949 (68.9)
N/a	1,033 (18.0)
**Origin**	
Antananarivo	1,032 (18.0)
Fianarantsoa	1,552 (27.1)
Mahajanga	1,009 (17.6)
Toamasina	1,032 (18.0)
Toliara	1,111 (19.4)

Environmental samples (water and soil) were later collected from areas with a high proportion of blood donors indicating exposure to Bp (Fig 1), in accordance with international Bp sampling guidelines [41] and as previously described [12]. Soil sampling involved collecting 20 samples per site at regular 5 m intervals in a cross pattern using a spade to excavate up to 30 cm depth. Approximately 30 g of soil per hole was transferred into labelled 15 × 10 cm Ziplock bags using a disinfected hand trowel. For water sampling, 1 L of surface water was collected in labelled sterile bottles while avoiding large debris. All equipment was thoroughly cleaned with water and 70% ethanol between samples to prevent cross-contamination. Samples were stored in insulated containers, kept cool and shaded, and promptly transported to the laboratory for processing under controlled conditions.

### Antigen selection

Antigens were selected using previous Luminex assays utilizing conjugated and purified Bp antigens [36,42]. Briefly, serum samples from culture-confirmed melioidosis patients collected in Australia were utilized to screen Luminex antigen reactivity. A subset of the six reactive antigens (GroEL, Heat Shock Protein 60; AhpC, Alkyl Hydroperoxide Reductase C; HCP1, Haemolysin Co-regulated Protein 1; CPS I, Capsular Polysaccharide type I; LPSA, Lipopolysaccharide A and LPSB, Lipopolysaccharide B) was selected for this study based on their demonstrated persistence in antibody responses up to 70 days post-admission, as reported by Settles et al. (2023) [36].

### Comparative sequence analysis of antigen specificity

To assess the antigenic specificity of the six selected antigens from Bp, pairwise sequence similarity searches were performed using the protein BLAST (BLASTp) algorithm against the NCBI non-redundant protein database [43]. The analysis included selected protein antigens (HCP1, AhpC, GroEL) as well as full sets of biosynthetic proteins, described by Holden MTG et al, from the CPS I and LPSA clusters [44].

Each query sequence was submitted to BLASTp with default parameters. The top-ranking homologs from other organisms were retrieved, and key parameters, E-value (expectation values) and amino acid identity, were recorded to evaluate similarity. E-values represent the number of matches expected to occur by chance in a database of a given size. Lower E-values indicate greater statistical significance of the match. E-values approaching zero suggest highly significant matches, while higher E-values (e.g., > 1e-5) suggest limited or no meaningful similarities.

Amino acid identity was used to quantify the proportion of residues that are identical between the query and subject sequences in the aligned region. High identity values (e.g., > 90%) indicate strong evolutionary conservation, often reflecting functional or structural similarity. Conversely, identity values below 40% generally indicate more distant homology or functional divergence, and are suggestive of antigenic specificity if present uniquely in the target organism.

Protein alignments and annotations of top hits were examined to determine whether homologs were found in pathogenic or environmental organisms, providing insights into the likely cross-reactivity of each antigen. This approach allowed us to rank antigens based on their degree of conservation and potential specificity for Bp.

### Luminex technique

Luminex beads were coupled with six selected and purified Bp antigens, as previously described [42] (Table 2). The protein antigens, each containing an N-terminal 6-histidine tag, were conjugated to distinct fluorescently labelled MagPlex microspheres using a two-step carbodiimide coupling reaction at pH 5.6 with Sulfo-NHS (N-hydroxysulfosuccinimide) and EDC (1-ethyl-3-(3-dimethylaminopropyl) carbodiimide hydrochloride), following the Luminex xMAP Cookbook (2nd Ed.). Each conjugation used 48 μg of protein per million beads. Successful conjugation was verified by detecting the 6-histidine tag using a monoclonal anti-6-histidine antibody (Abcam, ab15145) conjugated to biotin, with validation performed via a Luminex MAGPIX instrument. The purified carbohydrates, CPS I and LPS, were conjugated to the beads through activated carbohydrate (active ether) chemistry using 4-(4,6-dimethoxy-1,3,5-triazin-2-yl) -4-methyl-morpholinium chloride (DMTMM). Following activation with DMTMM, the reactive carbohydrate conjugates were purified using PD-10 desalting columns. Successful conjugation was confirmed using CPS I- and LPS-specific monoclonal antibodies kindly provided by Dr. David AuCoin (University of Nevada, Reno). This conjugation and assay setup followed the methodology described by Celona K.R. et al. (2023).

**Table 2:** Selected Bp antigens and controls used for Luminex Assay.

Antigen	Locus Tag	Sub-Localization^1^	Antigen Type
HCP1	BPSS1498	Extracellular	Protein
GroEL	BPSL2697	Cytoplasmic	Protein
AhpC	BPSL2096	Cytoplasmic	Protein
CPS I	N/A	Outer Membrane^3^	Carbohydrate
LPSA	N/A	Outer Membrane^2^	Carbohydrate
LPSB	N/A	Outer Membrane^2^	Carbohydrate
IgG Positive Control	N/A	N/A	N/A
EBV Positive Control	N/A	N/A	N/A
PE Instrument Control	N/A	N/A	N/A

N/A, Not Applicable; EBV, Epstein-Barr Virus; PE, phycoerythrin; ^1^PSORTb Subcellular Localization Predication Tool https://www.psort.org/psortb/; ^2^Perry, MacLean [45]; ^3^Masoud, Ho [46].

To ensure assay reliability, performance was assessed using the same reference serum set at Northern Arizona University and the Institut Pasteur of Madagascar, consisting of two sera from culture-confirmed Bp-positive individuals and one serum from a healthy individual from a non-endemic region (United States). Strong concordance in signal intensities between both laboratories (Pearson’s r = 0.86, p < 0.001) confirmed the robustness of the assay (Fig A in S1 Appendix). Each assay plate included positive and negative control sera for consistency, along with additional controls: beads conjugated with phycoerythrin (PE) alone to monitor instrument response, and beads coupled with anti-IgG antibodies to ensure proper binding of anti-IgG–PE antibodies. In total, 5,736 serum samples were analysed for reactivity to the six Bp antigens.

Briefly, serum was diluted 1000-fold and detected for IgG reactivity against Bp antigens. Serum samples were distributed into specific wells and incubated with Bp antigen coupled-MAGPLEX-microsphere beads (Luminex) coupled with the six antigens and PE. After 45 minutes of incubation, the magnetic microspheres were incubated for 60 seconds on a magnet plate and washed with assay buffer (PBS 0.05%, BSA 0.1%, tween pH 7.4). Following the washing step, a phycoerythrin-labeled conjugate (H10104, Thermofisher, Massachusetts, U.S.) recognizing IgG was added to the wells, forming an antigen-antibody-conjugate-PE complex. The excess conjugate was removed by washing, and the microplate was read using a Magpix instrument (MAGPX12234702, Texas, USA). The beads were identified, and PE fluorescence was quantified, with the fluorescence intensity being proportional to the quantity of specific antibodies in the sample.

### Threshold definition

In the absence of well-characterized Madagascar local samples to set positivity thresholds using standard methods (e.g., receiver operating characteristic curve or ROC), we applied a Gaussian Mixture Model (GMM) to classify subpopulations based on reactivity levels [47]. GMM treats continuous data as clusters, each following a normal distribution, categorizing individuals as non-reactive, low-reactive, or highly reactive. The optimal number of clusters may be assessed using the Goodness of Fit (GoF) test, which compares observed and predicted distributions, where a significantly different fit (p ≤ 0.05) indicates poor model fitness. For all six tested antigens, a significant GoF rejected the GMM model. We thus considered a single Gaussian distribution within the population. Consequently, the positivity threshold was set at the mean + 3 standard deviations across the dataset.

### Environmental DNA culture and extraction (Water and Soil Samples)

Soil and water samples were enriched in broth prior to DNA extraction as previously described [12]. Briefly, 20 g of soil or filtered debris from 1 L of water were cultured in 30 mL of Ashdown broth supplemented with colistin (50 mg/L) for 48 hours at 37°C under agitation. The culture supernatant was then collected, and DNA was extracted using the QIAamp Fast DNA Stool Mini Kit (QIAGEN, ref. 51604).

The extraction protocol involved adding 1 mL of Inhibitex buffer to 3 mL of the supernatant to remove potential inhibitors, followed by the addition of 15 µL of proteinase K and 200 µL of lysis buffer. The mixture was incubated at 70°C for 10 minutes. Subsequently, 200 µL of ethanol was added, and the lysate was applied to QIAamp spin columns for DNA purification. The columns were washed sequentially with 500 µL of AW1 and AW2 buffers, and DNA was eluted using 200 µL of ATE buffer.

The extracted DNA was quantified using a multiscan spectrophotometer and stored at -20°C until further use.

### Real-time PCR for the detection of Bp

Two real-time PCR assays were conducted. The first assay targeted the RNA16S gene to detect bacterial presence, using a primer pair designed with Primer-BLAST [48]. The primer sequences, RNA16S_fwd (GCCTTCGGGTTGTAAAGCAC) and RNA16S_R (ACCAATGCAGTTCCCAGGTT), amplify a 224-bp product.

The second assay targeted the type III secretion system (TTS1) of Bp, following the method described and validated by Novak et al. [49]. Briefly, a primer pair targeting the TTS gene cluster of Bp (GenBank accession no. AF074878) was selected and validated using Primer-BLAST. The primer sequences, BpTT4176F (5’-CGTCTCTATACTGTCGAGCAATCG-3’) and BpTT4290R (5’-CGTGCACACCGGTCAGTATC-3’), were designed to amplify a 115-bp product and the fluorogenic probe, BpTT4208P (5’- CCGGAATCTGGATCACCACCACTTTCC-3’) with a 6-carboxyfluorescein reporter molecule attached at the 5′ end and a Black Hole Quencher 1 on the 3′ end, to detect amplification of this specific region. Primers and probes were synthesized by Eurogentec (Belgium).

Each real-time PCR reaction was set up in a total volume of 25 µL, consisting of 5 µL of extracted DNA (diluted 10x if inhibitors were suspected) and 20 µL of master mix, which included 12.5 µL of either Applied Biosystems Power SYBR Green Universal Master Mix (Applied Biosystems, California, US) or LightCycler FastStart DNA Master Mix (Roche Diagnostics, Indianapolis, IN) for RNA16S and TTS1 respectively, 400 nM of each primer, and 200mM of probe (if applicable). Amplification and detection were carried out using the QuantStudio 5 Real-Time PCR System (Thermo Fisher Scientific, Waltham, MA, USA).

Thermal cycling conditions included an initial enzyme activation step at 95°C for 10 minutes, followed by 40 cycles of 95°C for 15 seconds and 59°C (RNA16S) or 60°C (TTS1) for 1 minute. The cycle threshold values represent the calculated cycles at which fluorescence from SYBR Green binding or the cleaved probe for RNA16S and TTS1 amplification respectively exceeds a fixed threshold, as calculated by the instrument for each reaction.

Each PCR run included a no-template control (PCR-grade water) to check for contamination, and a positive control (DNA extracted from Bp strains maintained by Rakotondrasoa A. et al. [21]) to confirm successful amplification and rule out potential amplification failures.

### Statistical analysis

Data analysis was performed using GraphPad Prism version 8.0 (GraphPad, La Jolla, CA). The Shapiro–Wilk test was used to assess the normality of the data distribution, guiding the choice of statistical tests. For non-normally distributed data, the Mann-Whitney test was used to compare medians between groups. For normally distributed data, the t-test was used. The Chi-square test was employed to compare positivity rates. Multiple testing corrections were made using the Bonferroni method, and p-values below 0.05 were considered significant. Principal component analysis (PCA) was performed with Rstudio (Version 4.3.2) to describe and visualize the dataset.

## Results and discussion

### Antigen-specific sequence conservation supports diagnostic value of selected *Burkholderia pseudomallei* (Bp) antigens

Comparative amino acid sequence analyses were conducted to evaluate conservation of the six Bp antigens employed in serodiagnosis. Protein BLAST searches against the NCBI non-redundant database were performed for both structural antigens (HCP1, GroEL, and AhpC) and biosynthetic enzymes associated with carbohydrate antigens (CPS I and LPS). Among these, HCP1 exhibited the lowest sequence identity (31.1–32.9%) with non-pathogenic *Burkholderia* species, underscoring its high specificity and diagnostic value. In contrast, GroEL and AhpC were highly conserved across the genus, with identity values between 94.5% and 100.0%, limiting their discriminative potential (Results 1–3 in S1 Appendix).

Analysis of the LPS biosynthesis pathway revealed substantial sequence conservation among several enzymes across *Burkholderia* and *Paraburkholderia* species, with identities ranging from 50.1% to 91.2%. Notably, *B. thailandensis*, *B. singularis*, and *Paraburkholderia domus* shared >90% identity for several enzymes, indicating partial conservation of this pathway among environmental or less-virulent relatives. However, distinct combinations and organizations of these enzymes could confer additional specificity in Bp, particularly in the context of pathogenesis (Result 4 in S1 Appendix).

Similarly, BLAST analysis of CPS I-associated biosynthetic proteins demonstrated high sequence identity (up to 90%) predominantly within closely related *Burkholderia* species, such as *B. seminalis*, *B. plantarii*, and *B. singularis*, with minimal homology outside the genus. Occasional moderate similarity was observed with more distantly related bacteria like *Candidatus* Hamiltonella defensa, though such instances were rare. These findings suggest that CPS I biosynthesis proteins in Bp are relatively specific, reinforcing their potential utility as serodiagnostic targets with limited risk of cross-reactivity. Nevertheless, in regions where non-pathogenic *Burkholderia* species are prevalent, potential cross-reactivity should be considered due to shared enzymatic determinants (Result 5 in S1 Appendix).

Together, the data highlight the high specificity of HCP1 and support the diagnostic value of CPS I in differentiating Bp infections (Table 3). These findings align with previous studies and provide a molecular basis for the selective use of these antigens in serological assays [50–53].

**Table 3:** Comparative sequence results of selected Bp antigens.

Antigen	Type	Specificity Metric	Closest Non-Bp Match	Notes
Hcp1	Protein	31.1–32.9% identity to various species	*Chitinasiproducens palmae*	High specificity*
AhpC	Protein	94.5–100.0% identity to *Burkholderia* spp.	*Burkholderia multivorans*	Low specificity* (conserved)
GroEL	Protein	98.5-99.6% identity to *Burkholderia* spp.	*Burkholderia thailandensis*	Low specificity* (conserved)
CPS I	Carbohydrate	Avg. 64.3% (34.3-81.5%) identity across 24 enzymes	*Burkholderia spp.*	3/24 enzymes S, 13/24 enzymes MS, 8/24 enzymes NS*
LPS	Carbohydrate	Avg. 74.4% (50.1-91.2%) identity across 17 enzymes	*Burkholderia spp.*	0/17 enzymes S, 7/17 enzymes MS, 10/17 enzymes NS*

(*)S, specific; MS, moderately specific; NS, non-specific (<40%, 40–70%, > 70% identity to non-Bp).

### Seroreactivity varies by antigen and geographic region

Antigen-specific seroreactivity showed marked geographic differences across the five regions of Madagascar. Among the six antigens tested, LPSA and LPSB elicited the highest median fluorescence intensity (MFI) values (p < 0.001), with median signals exceeding 2,300 in all regions. The strongest responses were observed for LPSB in Toamasina (MFI = 4,772.7) and LPSA in Antananarivo (MFI = 4,330.7), indicating their high immunogenic potential.

**Fig 2:**
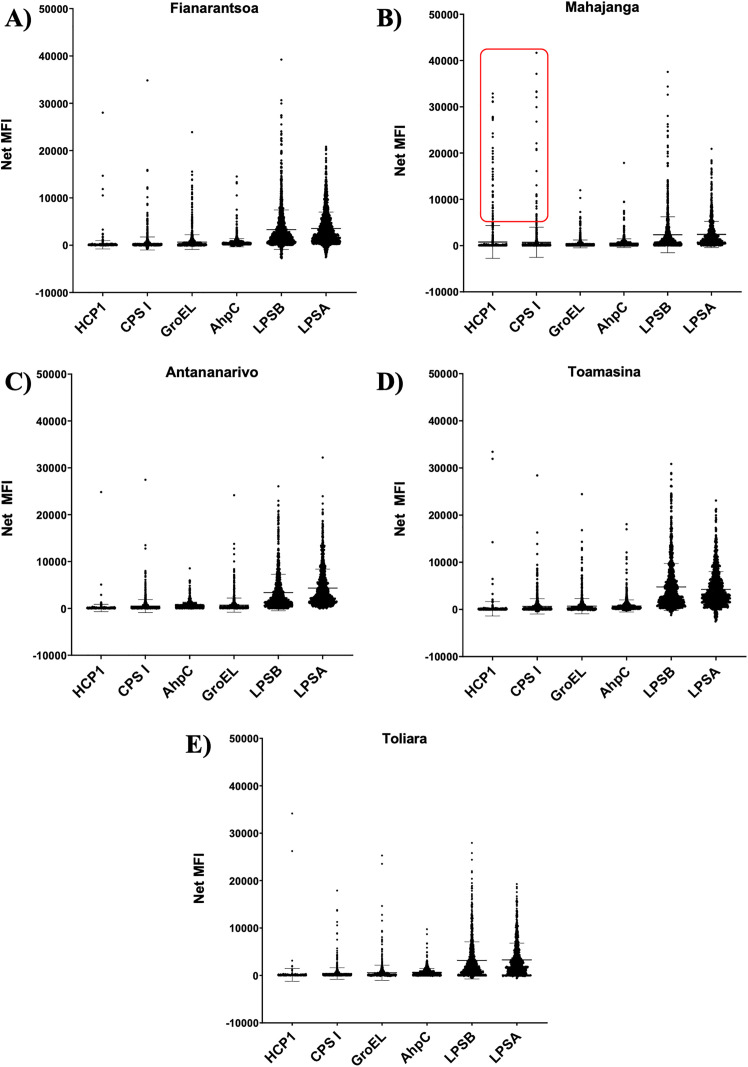
Population reactivity to the six *Burkholderia pseudomallei* antigens. Bars represent mean and standard deviation. A) Fianarantsoa (Southern highland). B) Mahajanga (West coast, Individuals with strong reactivity to HCP1 and CPS I are highlighted with a red rectangle), C) Antananarivo (Central highland), D) Toamasina (East coast), E) Toliara (South-west).

In contrast, Hcp1 and CPS I showed significantly lower MFIs (p < 0.0001), ranging from 90.3–154.3 for Hcp1 and 361.1–710.8 for CPS I, suggesting weaker antibody responses. Notably, Mahajanga displayed a tendency toward higher mean Hcp1 reactivity (MFI = 776.5), which may reflect recent or localized exposure, although this difference was not statistically significant (Fig 2).

Overall, these results indicate that LPS antigens are associated with strong, widespread antibody responses, possibly reflecting past or cumulative exposure, whereas Hcp1 may serve as a more specific marker of recent infection. This antigenic heterogeneity emphasizes the need to consider multiple antigens when interpreting serological data and inferring exposure patterns across regions.

### Antigen co-reactivity patterns and exposure classification

Principal component analysis (PCA) identified distinct co-reactivity patterns among Bp antigens. The first principal component (accounting for 55% of the total variance) was defined predominantly by CPS I, AhpC, and LPSA, suggesting markers of prolonged or past exposure. This observation aligns with findings by Suttisunhakul et al. (2016) and Pumpuang et al. (2017–2019), who reported that the strong immunogenicity and persistence of LPS and CPS antibody responses are characteristic of chronic or previous infections [33,52,32]. In contrast, the second component was driven primarily by Hcp1 and CPS I, indicative of more recent infection signatures (Fig B in S1 Appendix). This pattern is consistent with the results of Pumpuang et al. (2017) and Phokrai et al. (2018), who identified Hcp1 as a sensitive marker of early or acute infection [50,52]. Together, these findings highlight the antigenic heterogeneity among Bp antigens and emphasize the value of combining multiple markers for effective serological surveillance and stage-specific diagnosis.

Gaussian Mixture Modeling (GMM) further separated a largely unexposed population from a small high-reactivity cluster, likely representing true exposures. Defining an exposure threshold as mean + 3 SD supported this classification and identified individuals with significant antigen reactivity.

### Elevated seroprevalence and multi-antigen reactivity in Mahajanga

**Fig 3:**
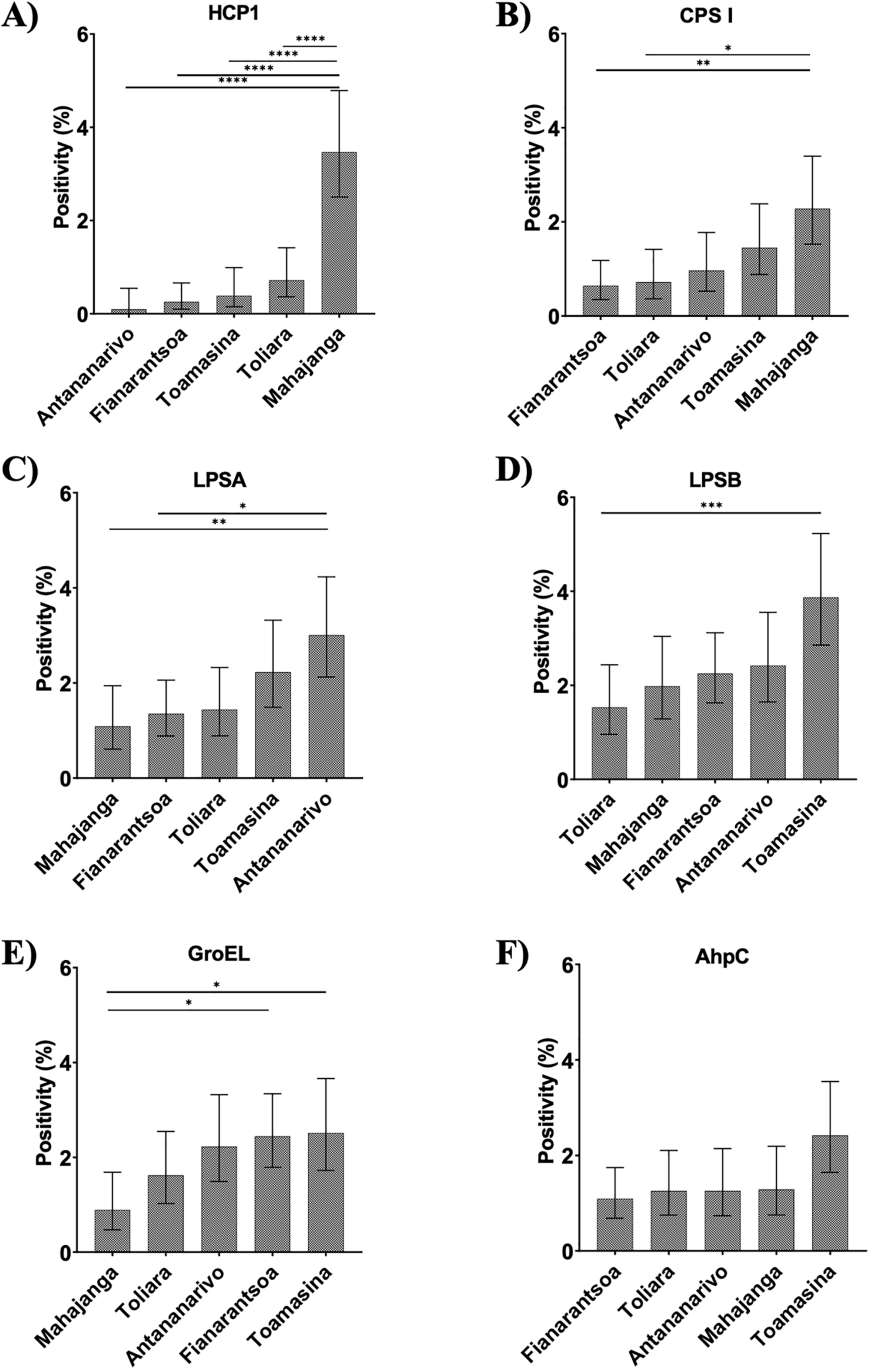
Population positivity per antigen based on locally determined cutoff in the 5 investigated regions. A) anti-HCP1, B) anti-CPS I, C) anti-LPSA, D) anti-LPSB, E) anti-GroEL, F) anti-AhpC antibody seropositivity. Chi-square test, « * », p = 0.04; « ** »,p = 0.001; « *** »,p = 0.0003; «**** »,p < 0.0001.

Among 5,736 serum samples analyzed, 6.7% (n = 385) were reactive to at least one antigen. Notably, 0.9% reacted to two antigens, and 0.3% to three or more. Importantly, only samples from Mahajanga showed reactivity to four or more antigens, with two individuals exhibiting responses to all six antigens (results shown in Table 4, in Fig 3 and as upSet plots in Fig C of S1 Appendix). These individuals were farmers, indicating professional environmental exposure was a likely route. However, no significant associations were found with occupation or gender (Table 5), suggesting environmental factors were the primary exposure risk. Mahajanga stood out with significantly higher HCP1 seroprevalence (3.5% vs. 0.1–0.7%, p < 0.0001) and increased odds of seropositivity (OR = 10.6, 95% CI: 6.6–17.4). Odds ratios were calculated by comparing each region individually against all remaining regions combined (i.e., Mahajanga vs. all-Mahajanga, Fianarantsoa vs. all-Fianarantsoa, Toamasina vs. all-Toamasina, Antananarivo vs. all-Antananarivo, and Toliara vs. all-Toliara), allowing for the identification of regions with distinct serological profiles. These results align with prior data showing Bp presence in Mahajanga soil and suggest endemic transmission [21].

**Table 4:** Cumulative antigens reactivity in each region (Nags) (threshold mean+3SD).

Regions	Antananarivo	Fianarantsoa	Mahajanga	Toamasina	Toliara	Overall
Nags	%	%	%	%	%	%
	N (95%CI)	N (95%CI)	N (95%CI)	N (95%CI)	N (95%CI)	N (95%CI)
6			0.2			0
			2 (0-0.7)			2 (0-0.1)
4			0.2			0
			2 (0-0.7)			2 (0-0.1)
3	0.2	0.5	0.5	0.1	0.1	0.3
	2 (0-0.7)	7 (0.2-0.9)	5 (0.2-1.2)	1 (0-0.5)	1 (0-0.5)	16 (0.2-0.5)
2	1	0.7	0.9	1.4	0.6	0.9
	10 (0.5-1.8)	11 (0.4-1.3)	9 (0.5-1.7)	14 (0.8-2.3)	7 (0.3-1.3)	51 (0.7-1.2)
1	7.5	5.3	5.9	9.9	5.8	6.7
	77 (6-9.2)	82 (4.3-6.5)	60 (4.6-7.6)	102 (8.2-11.8)	64 (4.5-7.3)	385 (6.1-7.4)
0	91.4	93.6	92.3	88.7	93.5	92.1
	943 (89.5-92.9)	1452(92.2-94.7)	931 (90.5-93.8)	915 (86.6-90.5)	1039 (91.9-94.8)	5280 (91.3-92.7)
Overall	100	100	100	100	100	100
	1032 (99.6-100)	1552 (99.8-100)	1009 (99.6-100)	1032 (99.6-100)	1111 (99.7-100)	5736 (99.9-100)

Nag, number of antigens reactivity; N, Number of reactive individuals; 95%CI, 95% confidence interval.

**Table 5:** Odds of HCP1 positivity associated with regional localization, gender, and occupation calculated using R, package epitools.

Variable	n(%)	OR	95%CI	Pvalue
**Origin**				
Antananarivo	1032(18.0)	0.2	0.0 - 0.5	0.00*
Fianarantsoa	1552(27.1)	0.2	0.1 - 0.5	0.00*
Mahajanga	1009(17.6)	**10.6**	**6.6-17.4**	0.00*
Toamasina	1032(18.0)	0.4	0.1 - 0.8	0.00*
Toliara	1111(19.4)	0.6	0.3 - 1.1	0.10
Mahajanga				
**Sex**				
Male	438(43.4)	0.8	0.3 - 2.2	0.5
Female	150(14.8)			
N/a	421(41.7)			
**Profession**				
Farmer	53(5.3)	0.9	0.3 - 6.4	0.9
Non Farmer	766(75.9)			
N/a	190(18.8)			

n(%), number of samples (percentage); OR, Odds ratio; 95%CI, 95% confidence interval;(*)significant P-value < 0,05.

### Detection of Bp in environmental water samples supports serological findings

**Fig 4:**
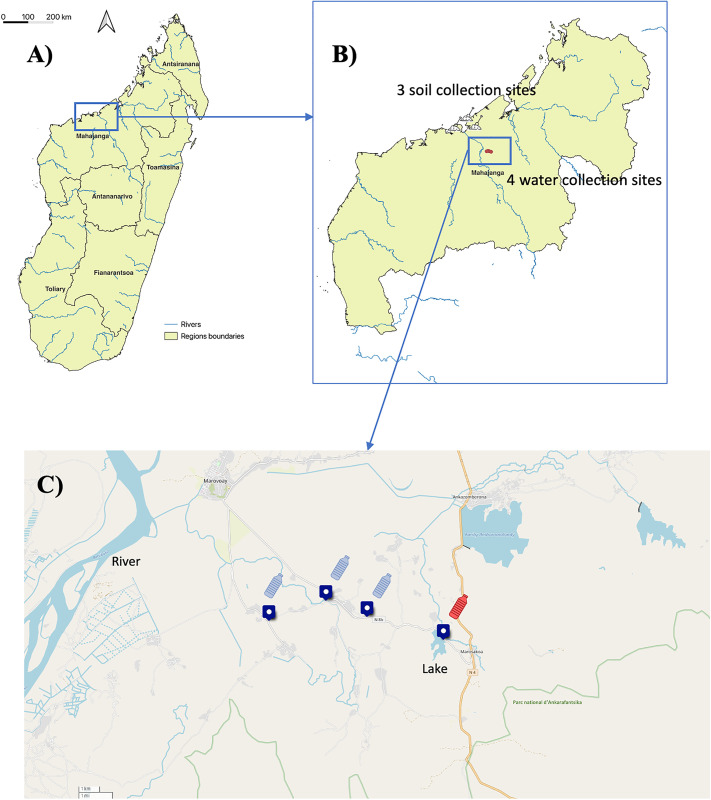
Map of Madagascar showing the sampling sites for sera and environmental samples. A) Map of Madagascar showing the regional blood transfusion centers in the five regions and the environmental sampling site B) Enlarged map of Mahajanga, highlighting the area where environmental samples were collected. Soil sampling was performed in three collection sites, each consisting of 20 soil samples spaced 5 meters apart per sampling point. Water samples collected from four different sites. One liter of water was collected per sampling point. C) Map of the four water collection points in Marovoay. Blue samples were RT-PCR Bp negative and the one represented in red was positive and Lake where the Bp positive sample was collected. Map created in QGIS using base layers from OpenStreetMap. Base map source: OpenStreetMap (public domain). Downloaded from [https://umap.openstreetmap.fr/en/map/new/#6/51.000/2.000]. Terms of use: [https://www.openstreetmap.org/copyright].

Prompted by the detection of multi-antigen-positive individuals, environmental sampling in Mahajanga and nearby Marovoay was initiated (Fig 4). Soil samples collected during the dry season and water samples collected during the rainy season were analyzed (Result 6 in S1 Appendix). While all soil samples were negative for TTS1 despite testing positive for RNA16S, one water sample tested positive for both, confirming the presence of Bp (Ct = 32.2 for TTS1). The expected 115 bp amplicon was confirmed via gel electrophoresis. These results corroborate human serological data and point to environmental exposure in Mahajanga, likely driven by seasonal dynamics. Similar findings in other endemic countries (e.g., India, Laos, Australia) emphasize the role of rainy seasons in bacterial dissemination through water and soil [54–58].

### Implications for surveillance and public health

This integrated analysis demonstrates the antigenic specificity and seroepidemiological utility of six selected Bp antigens in Madagascar. Among them, HCP1 and CPS I emerge as highly specific markers of recent or acute infections, while LPS antigens are indicative of long-term exposure. The high rates of seroprevalence and multi-antigen reactivity observed in Mahajanga point to localized endemicity and ongoing environmental transmission.

The detection of Bp in water sources during the rainy season confirms its seasonal environmental presence and underscores the importance of targeted environmental monitoring. This is particularly critical in the context of climate change, as Madagascar is experiencing increasingly frequent and intense cyclones. Such extreme weather events may exacerbate the environmental burden of melioidosis and contribute to a higher risk of transmission [59]. These findings highlight the urgent need to expand environmental sampling across the country, especially in the southeastern coastal regions, which have been repeatedly affected by cyclones and where sample collection remains challenging due to limited accessibility. The absence of significant associations with occupational exposure or sex suggests that surveillance efforts should prioritize geographic and environmental risk factors rather than demographic characteristics.

While our findings point toward genuine exposure, the possibility of serological cross-reactivity with closely related species, particularly *Burkholderia thailandensis* (Bt) and CPS-variant *Burkholderia* spp., must be considered when interpreting antibody responses, especially to LPS and CPS I antigens. Previous studies have shown that although the genes involved in LPS and O-polysaccharide biosynthesis differ between Bp and Bt, their structural and immunological features can be sufficiently conserved to elicit cross-reactive antibody responses [60–63]. Conversely, certain protein antigens such as Hcp1 tend to be more discriminatory and have demonstrated limited serological overlap [30,50].

Importantly, relying on a single antigenic marker can lead to misclassification, particularly in settings where environmental *Burkholderia* exposure is frequent. The integrated use of multiple, well-characterized antigens, as applied in this study with six independent Bp targets, enhances diagnostic specificity by capturing distinct immunological signatures and mitigating the impact of any single antigen’s potential cross-reactivity. This multi-antigen approach, when interpreted alongside molecular detection methods, offers a more robust framework for confirming Bp exposure and distinguishing it from serological background reactivity to non-pathogenic relatives.

In a surveillance context, this underscores the importance of combining broad-based serological panels with confirmatory molecular assays to minimize potential misclassification. Future work should therefore include empirical evaluation of cross-reactivity using local Bt isolates and continued refinement of multi-antigen diagnostic algorithms. Such integration will improve the specificity of melioidosis surveillance, strengthen early detection of emerging *Burkholderia* infections, and support targeted environmental and public-health interventions in Madagascar and beyond.

## Supporting information

S1 Appendix **Result 1.** Multiple sequence alignment of Hemolysin Co-Regulated Protein 1 of *Burkholderia pseudomallei* with top four most similar organisms identified by BLAST NCBI. **Result 2.** Multiple sequence alignment of Alkyl Hydroperoxide Reductase C of *Burkholderia pseudomallei* with top four most similar organisms identified by BLAST NCBI. **Result 3.** Multiple sequence alignment of GroEL, Heat Shock Protein 60 of *Burkholderia pseudomallei* with top four most similar organisms identified by BLAST NCBI. **Result 4.** Multiple sequence alignment of the 17 Lipopolisaccharide (LPS) biosynthesis proteins from *Burkholderia pseudomallei.* **Result 5.** Multiple sequence alignment of the 24 Capsular Polysaccharide (CPS) biosynthesis proteins from *Burkholderia pseudomallei*. **Result 6.** DNA concentration from enriched soil and water extracted samples measured using multiskan sky (Thermoscientific, Singapore). **Fig 1.** Correlation of median fluorescence intensities from reference samples tested at Northern Arizona University (Reference) and the Immunology of Infectious Diseases Unit, Pasteur Institute of Madagascar (Test). **Fig 2.** Principal component analysis of reactivity to the six antigens. **Fig 3.** UpSet plot depicting the intersection of seropositivity across the six *Burkholderia pseudomallei* antigens.
(PDF)
