# Supplementary material for: Evidence of populational Burkholderia pseudomallei exposure in Madagascar
Source: PLoS Negl Trop Dis. 2025 Nov 18;19(11):e0013419. doi: 10.1371/journal.pntd.0013419 (PMC12638028; doi:10.1371/journal.pntd.0013419)
Supplement: S1 Appendix — Result 1. Multiple sequence alignment of Hemolysin Co-Regulated Protein 1 of Burkholderia pseudomallei with top four most similar organisms identified by BLAST NCBI. Result 2. Multiple sequence alignment of Alkyl Hydroperoxide Reductase C of Burkholderia pseudomallei with top four most similar organisms identified by BLAST NCBI. Result 3. Multiple sequence alignment of GroEL, Heat Shock Protein 60 of Burkholderia pseudomallei with top four most similar organisms identified by BLAST NCBI. Result 4. Multiple sequence alignment of the 17 Lipopolisaccharide (LPS) biosynthesis proteins from Burkholderia pseudomallei. Result 5. Multiple sequence alignment of the 24 Capsular Polysaccharide (CPS) biosynthesis proteins from Burkholderia pseudomallei. Result 6. DNA concentration from enriched soil and water extracted samples measured using multiskan sky (Thermoscientific, Singapore). Fig 1. Correlation of median fluorescence intensities from reference samples tested at Northern Arizona University (Reference) and the Immunology of Infectious Diseases Unit, Pasteur Institute of Madagascar (Test). Fig 2. Principal component analysis of reactivity to the six antigens. Fig 3. UpSet plot depicting the intersection of seropositivity across the six Burkholderia pseudomallei antigens. (PDF) [file pntd.0013419.s001.pdf]

Full title : A hidden threat: First evidence of *Burkholderia pseudomallei* exposure in Madagascar
Short title : *Burkholderia pseudomallei* threat in Madagascar

Solohery Lalaina Razafimahatratra ; Sarobidy Tsiory Avotra Andriambinintsoa ; Lova Tsikiniaina Rasoloharimanana ; Minoarisoa Rajerison ; Voahangy Rasolofo ; Carina M. Hall ; Kimberly R. Celone ; Jinhee Yie ; Paul Keim ; David M. Wagner ; Erik W. Settles ; Matthieu Schoenhals

Contents of the appendix :

Result 1 : Multiple sequence alignment of Hemolysin Co-Regulated Protein 1 of *Burkholderia pseudomallei* with top four most similar organisms identified by BLAST NCBI……………………………………………..p.2

Result 2 : Multiple sequence alignment of Alkyl Hydroperoxide Reductase C of *Burkholderia pseudomallei* with top four most similar organisms identified by BLAST NCBI..……………………………………….p.3

Result 3 : Multiple sequence alignment of GroEL, Heat Shock Protein 60 of *Burkholderia pseudomallei* with top four most similar organisms identified by BLAST NCBI ……………………………………………p.4

Result 4 : Multiple sequence alignment of the 17 Lipopolisaccharide (LPS) biosynthesis proteins from *Burkholderia pseudomallei*..…………………………………………………..……………………………p.6

Result 5 : Multiple sequence alignment of the 24 Capsular Polysaccharide (CPS) biosynthesis proteins from *Burkholderia pseudomallei*..………………………………………………………………………………p.12

Result 6 : DNA concentration in soil and water extracted samples measured using multiscan..…...….p.16

Fig A : Correlation of median fluorescence intensities from reference samples tested at Northern Arizona University (Reference) and the Immunology of Infectious Diseases Unit, Pasteur Institute of Madagascar (Test)……………………………………………………..…………………………………..………….p18

Fig B : Principal component analysis of reactivity to the six antigens…..…………………………………p19

Fig C : UpSet plot depicting the intersection of seropositivity across the six *Burkholderia pseudomallei* antigens. ……………………………..…………………………………………………...……………………….p20

Result 1 : **Multiple sequence alignment of Hemolysin Co-Regulated Protein 1 of *Burkholderia pseudomallei* with top four most similar organisms identified by BLAST NCBI.** The alignment was performed using CLUSTAL Omega (1.2.4). Q63K67_BURPS : Hcp 1 protein of *Burkholderia pseudomallei* ; A0A939KDZ2 : Hcp family type VI secretion system effector of *Comamonas denitrificans* ; A0A1H2PL91 : Type VI secretion system secreted protein of *Chitinasiproducens palmae* ; A0A1H2PKZ8 : Type VI secretion system secreted protein of *Chitinasiproducens palmae* ; A0A1H2PKY4 : Type VI secretion system secreted protein of *Chitinasiproducens palmae*. "*",highly conserved residues ; ".",conserved in at least one sequence alignement ; ":", chemically similar residues ; "-" gap

```
[Q63K67_BURPS]      MLAGIYLKVKGKTQGEIKGSVVQEGHDGKIHILAFKNDYDMPARLQEGLTPAAAARGTIT
[A0A939KDZ2]        MPMPCYLMLEGQNQGKIEGSTKIQGHDGKILVQAVEHLVDIPKNPQTGLPAGKRVHQGIT
[A0A1H2PL91]        MPMPCYLTLEGQNQGKIEGSTKVQGHEGKILVQAVDHVIEIPKSPQTGLPTGKRVHGAMT
[A0A1H2PKZ8]        MPMPCYLTLEGQNQGKIEGSTKVQGHEGKIHVQAVDHTIEIPKSPQTGLPTGKRVHGAMT
[A0A1H2PKY4]        MPMPCYLTVEGQNQGKIEGSTKVQGHDGKIHVQAVNHTIEIPKSPQTGLPTGKRVHGAMV
                    *    ** ::*:.**:*:**.  :**:*** : *..:  ::*   * **  .   .: :.

[Q63K67_BURPS]      LTKEMDRSSPQFLQALGKREMMEEFEITIHRPKTDTTGGDLTELLFTYKFEKVLITHMDQ
[A0A939KDZ2]        LTKEVDKSSPKLFQALTSGEQMKSVVLEFYRISPKG----TEEKYYTITLSNAVIVSART
[A0A1H2PL91]        VTKEIDKSSPKLYQALTSGEQLKDVKLEFYRISPKG----TEEKYYTVKLENAILTSMKS
[A0A1H2PKZ8]        VTKEIDKSSPKLYQALTSGEQLKDVTLEFYRISPKG----TEEKYYTAKLENAILTNMKS
[A0A1H2PKY4]        VTKEIDKSSPKLYQALTSGEQLKDVTLEFYRISPKG----TEEKYYTAKLENAILTNMKS
                    :***:*:***:: *** . * ::.. : ::* . .       *  :* .:.:.::.

[Q63K67_BURPS]      YSPTPHKDDSNGIKEGLLGYIEEIKFTYSGYSLEHAESGIAGAANWTNG--
[A0A939KDZ2]        WVPNTLDPN-----FKQMGHMEDIGITYEKIVWTWVPDGIEAEDSWLAPKA
[A0A1H2PL91]        WTPNCLDPN-----NRQMGHMEDLAFTYEKITWTYEPDGIEAEDSWLAPKA
[A0A1H2PKZ8]        WTPNCLDPN-----NKQMGHMEDLAFTYEKITWTYEPDGIEAEDSWLAPKA
[A0A1H2PKY4]        WTPNCLDPN-----NRQMGHMEDLAFTYEKITWTYEPDGIEAEDSWLAPKA
                    : *.  . :        :*::*:: :**.        .** .  .*
```

Result 2 : **Multiple sequence alignment of Alkyl Hydroperoxide Reductase C of *Burkholderia pseudomallei* with top four most similar organisms identified by BLAST NCBI.** The alignment was performed using CLUSTAL Omega (1.2.4). Q63N06 _BURPS : Alkyl hydroperoxide reductase C of *Burkholderia pseudomallei* ; A0A103DW05 : Alkyl hydroperoxide reductase C of *Burkholderia singularis* ; A0A372JX48 : Alkyl hydroperoxide reductase C of *Paraburkholderia sp.* ; A0A658QWQ8 : Alkyl hydroperoxide reductase C of *Caballeronia concitans* ; A0A4R6F8N7 : Alkyl hydroperoxide reductase C of *Paraburkholderia sp.*. "*", highly conserved residues ; ".", conserved in at least one sequence alignement ; ":", chemically similar residues ; "-" gap

```
tr|Q63N06|Q63N06_BURPS              MPIINSPIKPFKATAYHNGDFVQVSDETLKGKWSVVVFYPADFTFVCPTE
tr|A0A103DW05|A0A103DW05_9____      MPIINTQIKPFKATAYHNGDFVPVSDETLKGKWSVVVFYPADFTFVCPTE
tr|A0A372JX48|A0A372JX48_9____      MPIINSQIKPFKATAYHNGDFQTVTDETLKGKWSVVVFYPADFTFVCPTE
tr|A0A4R6F8N7|A0A4R6F8N7_9____      MPIINSQIKPFKATAYHNGDFVPVTEENFKGKWSVVVFYPADFTFVCPTE
tr|A0A658QWQ8|A0A658QWQ8_9____      MPIINSQVKPFKATAYHNGDFVTVTDETLKGKWSVFVFYPADFTFVCPTE
                                    *****: :*************  *::*.:******.**************

tr|Q63N06|Q63N06_BURPS              LGDLAERYAEFQKLGVEIYAVSTDTHFTHKAWHDTSDTIAKIKYPMIGDP
tr|A0A103DW05|A0A103DW05_9____      LGDLADRYAEFQKLGVEIYAVSTDTHFTHKAWHDTSDTISKIQYPMIGDP
tr|A0A372JX48|A0A372JX48_9____      LGDLADRYAEFQKLGVEIYSVSTDTHFTHKAWHDTSDTIQKIKYPMIADP
tr|A0A4R6F8N7|A0A4R6F8N7_9____      LGDLADRYAEFQKLGVEIYSVSTDTHFTHKAWHDTSDTIQKIKYPMIADP
tr|A0A658QWQ8|A0A658QWQ8_9____      LGDLADRYDEFKKLGVEIYGVSTDTHFTHKAWHDTSDTISKIKYPMIGDP
                                    *****:** **:*******.******************* **:****.**

tr|Q63N06|Q63N06_BURPS              TLAISRNFDVLIEEEGLALRGTFVINPQGEIKLCEIHDNGIGRDAGELLR
tr|A0A103DW05|A0A103DW05_9____      TLMISRNFDVLIEEEGMALRGTFVINPEGEIKLCEIHDNGIGRDAGELLR
tr|A0A372JX48|A0A372JX48_9____      TLAISRNFDVLIEEEGMALRGTFVINPEGEIKLCEIHDNGIGRDAGELLR
tr|A0A4R6F8N7|A0A4R6F8N7_9____      TLAISRNFDVLIEEEGLALRGTFVINPEGEIKLCEIHDNGIGRDAGELLR
tr|A0A658QWQ8|A0A658QWQ8_9____      TLAISRNFDVLIEEEGLALRGTFVINPEGEIKLCEIHDNGIGRDAGELLR
                                    ** *************:**********:**********************

tr|Q63N06|Q63N06_BURPS              KVQAAQYVAAHPGEVCPAKWTPGADTLTPSLDLIGKI
tr|A0A103DW05|A0A103DW05_9____      KVQAAQYIAAHPGEVCPAKWTPGADTLTPSLDLIGKI
tr|A0A372JX48|A0A372JX48_9____      KVQAAQYIAAHPGEVCPAKWTPGADTLTPSLDLVGKI
tr|A0A4R6F8N7|A0A4R6F8N7_9____      KVQAAQYIAAHPGEVCPAKWTPGAETLTPSLDLIGKI
tr|A0A658QWQ8|A0A658QWQ8_9____      KVQAAQYVASHPGEVCPAKWTPGAETLTPSLDLIGKI
                                    *******:*:**************:********:***
```

Result 3 : **Multiple sequence alignment of GroEL, Heat Shock Protein 60 of *Burkholderia pseudomallei* with top four most similar organisms identified by BLAST NCBI.** The alignment was performed using CLUSTAL Omega (1.2.4). Q3JGX2 : Chaperonin GroEL of *Burkholderia pseudomallei* ; Q2T3W7 : Chaperonin GroEL of *Burkholderia thailandensis*; A0A124P7Z5 : Chaperonin GroEL of *Burkholderia singularis.* ; A0A1H7S760 : Chaperonin GroEL of *Paraburkholderia caballeronis* ; A0A940SFG7 : Chaperonin GroEL of *Paraburkholderia sp.* "*", highly conserved residues ; ".", conserved in at least one sequence alignement ; ":", chemically similar residues ; "-" gap

```
sp|Q3JGX2|CH602_BURP1               MAAKEIIFHDGARAKLVEGVNLLANAVKVTLGPKGRNVVLERSFGSPVVT
sp|Q2T3W7|CH602_BURTA               MAAKEIIFHDGARTKLVEGVNLLANAVKVTLGPKGRNVVLERSFGSPVVT
tr|A0A1H7S760|A0A1H7S760_9____      MAAKEIIFSDGARAKLVEGVNILANAVKVTLGPKGRNVVIERSFGSPVVT
tr|A0A124P7Z5|A0A124P7Z5_9____      MAAKEIIFHDGARSKLVEGVNLLANAVKVTLGPKGRNVVLERSFGSPVVT
tr|A0A940SFG7|A0A940SFG7_9____      MAAKEVAFSDHARSKLVEGVNLLANAVKVTLGPKGRNVVLERSFGSPIVT
                                    *****: * * **:*******:*****************:*******:**

sp|Q3JGX2|CH602_BURP1               KDGVSVAKEIELADKVQNIGAQLVKEVASKTSDAAGDGTTTATVLAQAIV
sp|Q2T3W7|CH602_BURTA               KDGVSVAKEIELADKVQNIGAQLVKEVASKTSDAAGDGTTTATVLAQAIV
tr|A0A1H7S760|A0A1H7S760_9____      KDGVSVAKEIELADKVQNIGAQLVKEVASKTSDLAGDGTTTATVLAQAIV
tr|A0A124P7Z5|A0A124P7Z5_9____      KDGVSVAKEIELADKVQNIGAQLVKEVASKTSDAAGDGTTTATVLAQAIV
tr|A0A940SFG7|A0A940SFG7_9____      KDGVSVAKEIELADKLQNIGAQLVKEVASKTSDAAGDGTTTATVLAQAIV
                                    ****************:**************** ****************

sp|Q3JGX2|CH602_BURP1               REGQKYVAAGLNPLDLKRGIDKAVAAAVDELKKISKPTTTSKEIAQVATI
sp|Q2T3W7|CH602_BURTA               REGQKYVAAGLNPLDLKRGIDKAVAAAVEELKKISKPTTTSKEIAQVATI
tr|A0A1H7S760|A0A1H7S760_9____      REGQKYVAAGLNPLDLKRGIDKAVIAAIEELKKISKPTTTSKEIAQVATI
tr|A0A124P7Z5|A0A124P7Z5_9____      REGQKYVAAGLNPLDLKRGIDKAVAAAVEELKKISKPTTTSKEIAQVATI
tr|A0A940SFG7|A0A940SFG7_9____      REGQKYVAAGLNPLDLKRGIDKAVIAAVEELKKISKPSTTSKEIAQVATI
                                    ************************ **::********:************

sp|Q3JGX2|CH602_BURP1               SANGEESIGQRIAEAIDRVGKEGVITVEDGKSLADELDVVEGLQFDRGYL
sp|Q2T3W7|CH602_BURTA               SANGEESIGQRIAEAIDRVGKEGVITVEDGKSLADELDVVEGLQFDRGYL
tr|A0A1H7S760|A0A1H7S760_9____      SANGEVSIGQRIAEAIDRVGKEGVITVEDGKSLADELDVVEGLQFDRGYL
tr|A0A124P7Z5|A0A124P7Z5_9____      SANGEESIGQRIAEAIDRVGKEGVITVEDGKSLADELDVVEGLQFDRGYL
tr|A0A940SFG7|A0A940SFG7_9____      SANGEESIGQRIAEAIDRVGKEGVITVEDGKSLADELDVVEGLQFDRGYL
                                    ***** ********************************************

sp|Q3JGX2|CH602_BURP1               SPYFINHPERQLAVLDEPFILLHDKKISNIRDLLPVLEQVAKAGRPLLIV
sp|Q2T3W7|CH602_BURTA               SPYFINNPDRQLAVLDEPFILLHDKKISNIRDLLPVLEQVAKAGRPLLIV
tr|A0A1H7S760|A0A1H7S760_9____      SPYFINNPDRQIAVLDDPYILLHDKKVSNIRDLLPVLEQVAKAGRPLLII
tr|A0A124P7Z5|A0A124P7Z5_9____      SPYFINNQDRQLAVLDEPYILLHDKKVSNIRDLLPVLEQVAKAGRPLLII
tr|A0A940SFG7|A0A940SFG7_9____      SPYFINNQDRQLAVLEEPFILLHDKKVSNIRDLLPVLEQVAKSGRPLLII
                                    ******: :**:***::*:*******:***************:******:

sp|Q3JGX2|CH602_BURP1               AEDVEGEALATLVVNNIRGILKTVAVKAPGFGDRRKALLEDIAILTGGQV
sp|Q2T3W7|CH602_BURTA               AEDVEGEALATLVVNNIRGTLKTVAVKAPGFGDRRKALLEDIAILTGGQV
tr|A0A1H7S760|A0A1H7S760_9____      AEDVEGEALATLVVNNIRGILKTVAVKAPGFGDRRKALLEDIAILTGGQV
tr|A0A124P7Z5|A0A124P7Z5_9____      AEDVEGEALATLVVNNIRGILKTVAVKAPGFGDRRKALLEDIAILTGGQV
tr|A0A940SFG7|A0A940SFG7_9____      AEDVEGEALATLVVNNIRGILKTVAVKAPGFGDRRKALLEDIAILTGGQV
                                    ******************* ******************************

sp|Q3JGX2|CH602_BURP1               ITEETGLTLEKATLQELGRAKRIEVGKENTTLIDGAGDKPNIDARVKQIR
sp|Q2T3W7|CH602_BURTA               IAEETGLTLEKATLQELGRAKRIEVGKENTTLIDGAGDKPNIDARVKQIR
tr|A0A1H7S760|A0A1H7S760_9____      IAEETGLTLDKATLAELGQAKRIEVGKENTTVIDGAGDPKNIEARVKQIR
tr|A0A124P7Z5|A0A124P7Z5_9____      IAEETGLTLEKATLAELGRAKRVEVGKENTTVIDGAGEKVNIEARVKQIR
tr|A0A940SFG7|A0A940SFG7_9____      IAEETGLTLEKATLAELGQAKRIEVGKENTTVIDGAGEKVNIEARVKQIR
                                    *:*******:**** ***:***:********:*****:  **:*******

sp|Q3JGX2|CH602_BURP1               AQIAEATSDYDREKLQERVAKLAGGVAVIKVGGATEVEVKEKKDRVDDAL
sp|Q2T3W7|CH602_BURTA               AQIADATSDYDREKLQERVAKLAGGVAVIKVGGATEVEVKEKKDRVDDAL
tr|A0A1H7S760|A0A1H7S760_9____      VQIEEATSDYDREKLQERVAKLAGGVAVIKVGGATEIEVKEKKDRVDDAL
tr|A0A124P7Z5|A0A124P7Z5_9____      VQIDEATSDYDREKLQERVAKLAGGVAVIKVGGATEVEVKEKKDRVDDAL
tr|A0A940SFG7|A0A940SFG7_9____      AQIAEATSDYDREKLQERVAKLAGGVAVIKVGGATEIEVKEKKDRVDDAL
                                    .** :*******************************:*************

sp|Q3JGX2|CH602_BURP1               HATRAAVEEGIVPGGGVALIRVKQAIAALAGANADQKAGISIVLRALEEP
sp|Q2T3W7|CH602_BURTA               HATRAAVEEGIVPGGGVALIRVKQAIAELTGANVDQKAGINIVLRALEEP
tr|A0A1H7S760|A0A1H7S760_9____      HATRAAVEEGIVPGGGVALIRVKQAISELTGANRDQDAGIKIVLRALEEP
tr|A0A124P7Z5|A0A124P7Z5_9____      HATRAAVEEGIVPGGGVALIRVKQAISSLTGVNADQKAGINIVLRALEEP
tr|A0A940SFG7|A0A940SFG7_9____      HATRAAVEEGIVPGGGVALIRVKQAIAELKGANADQNAGINIVRRALEEP
                                    **************************: * *.* **.***.** ******

sp|Q3JGX2|CH602_BURP1               LRQIVANAGEEASVVVATVAAGQGNYGYNAATGEYGDLVESGVLDPTKVT
```

```
sp|Q2T3W7|CH602_BURTA              LRQIVANAGEEASVVVATVAAGSGNYGYNAATGEYGDLVESGVLDPTKVT
tr|A0A1H7S760|A0A1H7S760_9____     LRQIVANAGEEASVVVAKVAAGTGNFGYDAATGKYGDLVESGVLDPTKVT
tr|A0A124P7Z5|A0A124P7Z5_9____     LRQIVTNAGEEASVVVAKVADGQGNFGYNAATGEYGDLVESGVLDPTKVT
tr|A0A940SFG7|A0A940SFG7_9____     LRQIVANAGEEASVIVAKVAEGSGNYGYNAATGQYGDLVETGVVDPTKVT
                                   *****:********:**.** * **:**:****:******:**:******

sp|Q3JGX2|CH602_BURP1              RTALQNAASIAGLLLTTDATVHEAPKDAPPAAPAGVPGAGGPGFDF
sp|Q2T3W7|CH602_BURTA              RTALQNAASIAGLLLTTDATVHEAPKDAPPAQPAGVPGAGGTGFDF
tr|A0A1H7S760|A0A1H7S760_9____     RTALQNAASVAGLLLTTDATVHEAPKDAQPAAPAGGPGGAGGGFDF
tr|A0A124P7Z5|A0A124P7Z5_9____     RTALQNAASIAGLLLTTDATVHEAPKDAPPAPPAGGPGAGGPGFDF
tr|A0A940SFG7|A0A940SFG7_9____     RTALQNAASVASLLLTTDATVHEAPKDAAPAPAPGGPGAGGPGFDF
                                   *********:*.**************** ** ..* **..* ****
```

Result 4 : **Multiple sequence alignment of the 17 Lipopolisaccharide (LPS) biosynthesis proteins from *Burkholderia pseudomallei*.** Top five homologous sequences for each protein were identified using BLAST searches against the NCBI non-redundant (nr) protein database, excluding *Burkholderia pseudomallei*. Uniprot ID, unique identifier of the protein in the UniProt database ; DB, source database from which the sequence information was retrieved ; Accession, accession number of the matched sequence from BLAST results ; Organism, species from which the matched sequence originates ; Length, total number of amino acids in the matched protein ; Score (Bits), alignment score reflecting the quality of the BLAST match : higher scores indicate better matches ; Identities (%), percentage of amino acids that are identical between the query and subject sequences ; Positives (%), percentage of amino acids that are either identical or similar (conservative substitutions ; E(), expectation value indicating the likelihood of the match occurring by chance; lower values denote more significant matches.

Uniprot ID	DB	Accession	Organism	Length	Score(Bits)	Identities(%)	Positives(%)	E()
BPSL2687	SP	Q2SYI5	*Burkholderia thailandensis* (strain ATCC 700388 / DSM 13276 / CCUG 48851 / CIP 106301 / E264)	282	1442	95,7	97,2	0
BPSL2687	TR	A0A124P853	*Burkholderia singularis*	282	1275	84	90,1	3,7E-176
BPSL2687	TR	A0A0B6RPH5	*Burkholderia plantarii*	283	1216	81	89,4	3,7E-167
BPSL2687	TR	A0A063BEH8	*Burkholderia sp,* lig30	278	1174	78,5	86,5	7,7E-161
BPSL2687	TR	F2L7U4	*Burkholderia gladioli* (strain BSR3)	282	1183	77,7	85,5	3,8E-162
BPSL2687	TR	A0A8A8D495	*Burkholderia seminalis*	279	1155	76,7	86,7	6,3E-158
BPSL2687	TR	A0A0H3KCR9	*Burkholderia multivorans* (strain ATCC 17616 / 249)	278	1121	75,1	86,2	9,1E-153
BPSL2687	TR	A0A2N7WCN1	*Trinickia soli*	284	1112	74	82,3	2,7E-151
BPSL2687	TR	A0A4U1IDQ9	*Trinickia terrae*	282	1128	73,2	83,2	9E-154
BPSL2687	TR	A0A2N7VFF2	*Trinickia dabaoshanensis*	284	1092	72,5	81,1	2,9E-148
BPSL2687	TR	A0A3D8JSJ4	*Trinickia dinghuensis*	284	1094	71,5	82,7	1,5E-148
BPSL2687	TR	A0A494XRZ8	*Trinickia fusca*	282	1103	71,3	81,9	5,8E-150
BPSL2687	TR	A0A1X7CHT3	*Trinickia caryophylli*	282	1052	71,1	82,2	3,4E-142
BPSL2687	TR	A0A1Q8J210	*Burkholderia sp,* SRS-W-2-2016	290	1051	70,8	82,5	6,3E-142
BPSL2687	TR	A0AAJ2E1G5	*Caballeronia sp,* LZ002	288	1038	70,4	81,5	5,6E-140
BPSL2687	TR	A0A6J5FQ04	*Paraburkholderia fynbosensis*	290	1048	69,9	80,8	1,8E-141
BPSL2687	TR	A0A1X7KTR0	*Paraburkholderia susongensis*	290	1044	69,7	81,8	7,4E-141
BPSL2687	TR	A0A0L0MFF8	*Candidatus Burkholderia verschuerenii*	283	1052	69,6	81,5	3,5E-142
H7C734	TR	A0A118DM62	*Burkholderia singularis*	353	1791	94,3	96,3	0
H7C734	TR	A0A9X1UP73	*Paraburkholderia tagetis*	353	1763	92,6	94,6	0
H7C734	TR	A0A372K139	*Paraburkholderia sp,* DHOC27	353	1750	91,8	94,1	0
H7C734	TR	A0A1H7R4H9	*Paraburkholderia caballeronis*	353	1756	91,5	94,9	0
H7C734	TR	A0A1X7KT42	*Paraburkholderia susongensis*	353	1751	91,5	94,9	0
H7C734	TR	B1FXM7	*Paraburkholderia graminis* (strain ATCC 700544 / DSM 17151 / LMG 18924 / NCIMB 13744 / C4D1M)	353	1747	91,5	94,1	0
H7C734	TR	A0A0H3KCH8	*Burkholderia multivorans* (strain ATCC 17616 / 249)	353	1745	91,5	94,6	0
H7C734	TR	A0A0B6RSX3	*Burkholderia plantarii*	353	1742	91,5	94,6	0
H7C734	TR	A0A1Q8J216	*Burkholderia sp,* SRS-W-2-2016	353	1755	91,2	94,9	0
H7C734	TR	A0A158FJ43	*Caballeronia choica*	353	1739	91,2	93,8	0
H7C734	TR	A0A6J5FRQ6	*Paraburkholderia fynbosensis*	353	1744	90,9	94,1	0
H7C734	TR	A0A6J5BVG8	*Paraburkholderia sediminicola*	353	1741	90,9	94,1	0
H7C734	TR	A0A940NEV7	*Paraburkholderia sp,* LEh10	353	1740	90,9	94,1	0
H7C734	TR	A0A7Z2JES4	*Paraburkholderia acidisoli*	353	1737	90,9	95,2	0

H7C734	TR	A0A9N8QVM7	*Paraburkholderia domus*	353	1737	90,9	94,1	0
H7C734	TR	A0A4U1IE72	*Trinickia terrae*	353	1736	90,9	93,8	0
H7C734	TR	A0A1N7RMB9	*Paraburkholderia ribeironis*	353	1742	90,7	94,1	0
H7C734	TR	A0A1H0ZY42	*Paraburkholderia fungorum*	353	1734	90,7	93,2	0
H7C734	TR	A0A1N6LGU5	*Paraburkholderia phenazinium*	353	1733	90,4	93,8	0
H7C734	TR	A0A6S7B667	*Paraburkholderia ultramafica*	353	1729	90,4	92,9	0
H7C734	TR	A0A4R6EZV3	*Paraburkholderia sp,* BL10I2N1	353	1727	90,4	93,5	0
H7C734	TR	A0A7Z2G2Q9	*Paraburkholderia acidiphila*	353	1719	90,3	93,8	0
H7C734	TR	A0A149Q0Z5	*Paraburkholderia monticola*	353	1733	90,1	94,1	0
H7C734	TR	A0A1H7DS06	*Paraburkholderia diazotrophica*	353	1732	90,1	94,6	0
H7C734	TR	F2L7U5	*Burkholderia gladioli* (strain BSR3)	353	1729	90,1	93,8	0
H7C734	TR	A0A370MZG1	*Paraburkholderia lacunae*	353	1728	90,1	93,5	0
H7C734	TR	A0A5B0GGR3	*Paraburkholderia panacisoli*	353	1726	90,1	93,5	0
H7C734	TR	Q144P4	*Paraburkholderia xenovorans* (strain LB400)	350	1711	90	93,1	0
H7C734	TR	A0A4R0XG40	*Paraburkholderia steynii*	353	1727	89,8	93,8	0
H7C734	TR	G4M6F2	*Candidatus Paraburkholderia kirkii* UZHbot1	353	1710	89,8	93,5	0
H7C740	SP	Q2SYH7	*Burkholderia thailandensis* (strain ATCC 700388 / DSM 13276 / CCUG 48851 / CIP 106301 / E264)	363	1817	97,8	98,9	0
H7C740	TR	A0A9N8MVY9	*Paraburkholderia domus*	363	1603	85,1	89,8	0
H7C740	TR	A0A512DDB4	*Cellulomonas aerilata*	351	1113	63,2	75,4	4,6E-149
H7C740	TR	A0A0Q5E8M1	*Plantibacter sp,* Leaf314	361	1129	62,7	75,1	2,4E-151
H7C740	TR	A0A5B2TCH1	*Pseudoroseomonas oryzae*	351	1114	62	74,1	3,2E-149
H7C742	SP	Q2SYI1	*Burkholderia thailandensis* (strain ATCC 700388 / DSM 13276 / CCUG 48851 / CIP 106301 / E264)	298	1472	95,3	97,7	0
H7C742	TR	A0A9N8MNL4	*Paraburkholderia domus*	300	1204	76,1	85,9	8,7E-165
H7C742	TR	A0A1N6N4Z8	*Aromatoleum tolulyticum*	297	951	63,7	73,6	2,4E-126
H7C742	TR	A0A0B6RIU9	*Burkholderia plantarii*	297	932	62,7	74,2	1,9E-123
H7C742	TR	Q5P5N2	*Aromatoleum aromaticum* (strain DSM 19018 / LMG 30748 / EbN1)	298	927	61,4	73,9	1,1E-122
H7C742	TR	A0A1X7CI37	*Trinickia caryophylli*	297	917	61,1	74,3	3,5E-121
H7C742	TR	A0A2T4IDS9	*Pseudothauera lacus*	300	920	60,3	74,2	1,4E-121
H7C759	TR	A0A9N8MPC4	*Paraburkholderia domus*	431	1727	79	87,5	0
H7C759	TR	F1W2P0	*Oxalobacteraceae bacterium* IMCC9480	286	767	61,2	75,2	4,7E-96
H7C759	TR	A0A261TZ14	*Bordetella genomosp,* 5	442	1353	60,6	74,6	0
H7C759	TR	A0A853FVK4	*Parapusillimonas granuli*	444	1373	59,8	74,7	0
H7C760	TR	A0A103DX19	*Burkholderia singularis*	297	1476	95,6	97,3	0
H7C760	TR	B2JFC5	*Paraburkholderia phymatum* (strain DSM 17167 / CIP 108236 / LMG 21445 / STM815)	297	1430	92,2	95,9	0
H7C760	TR	A0A1H7DMI2	*Paraburkholderia diazotrophica*	297	1420	91,6	95,3	0
H7C760	TR	A0A8A8D463	*Burkholderia seminalis*	297	1425	90,9	95,3	0
H7C760	TR	A0A4U1IDQ8	*Trinickia terrae*	297	1424	90,9	96,3	0
H7C760	TR	F2L7U6	*Burkholderia gladioli* (strain BSR3)	297	1420	90,9	94,3	0
H7C760	TR	A0A6J5FMP2	*Paraburkholderia fynbosensis*	297	1413	90,9	94,3	0
H7C760	TR	B1FXM6	*Paraburkholderia graminis* (strain ATCC 700544 / DSM 17151 / LMG 18924 / NCIMB 13744 / C4D1M)	297	1408	90,9	94,3	0
H7C760	TR	A0A494XJH4	*Trinickia fusca*	297	1415	90,2	94,9	0
H7C760	TR	A0A1N7RMA3	*Paraburkholderia ribeironis*	297	1405	90,2	93,9	0
H7C760	TR	A0A1H0ZZ75	*Paraburkholderia fungorum*	297	1404	90,2	93,6	0

H7C760	TR	A0A940NLB5	*Paraburkholderia sp,* LEh10	297	1399	90,2	93,9	0
H7C760	TR	A0A370MZJ7	*Paraburkholderia lacunae*	297	1404	89,9	94,3	0
H7C760	TR	A0A1Q8J219	*Burkholderia sp,* SRS-W-2-2016	297	1399	89,9	93,6	0
H7C760	TR	A0A7Z2GFZ8	*Paraburkholderia acidisoli*	297	1408	89,6	95,6	0
H7C760	TR	A0A0H3KH35	*Burkholderia multivorans* (strain ATCC 17616 / 249)	297	1407	89,6	96	0
H7C760	TR	A0A4R6EZM8	*Paraburkholderia sp,* BL10I2N1	297	1403	89,6	94,9	0
H7C760	TR	A0A0B6RW37	*Burkholderia plantarii*	297	1398	89,6	93,6	0
H7C760	TR	A0A6S7AX69	*Paraburkholderia ultramafica*	297	1391	89,6	93,3	0
H7C760	TR	A0AAJ1ZVZ5	*Caballeronia sp,* LZ002	297	1387	89,2	93,3	0
H7C760	TR	A0A1X7KUY1	*Paraburkholderia susongensis*	297	1395	88,9	93,9	0
H7C760	TR	A0A6J5BYW6	*Paraburkholderia sediminicola*	297	1394	88,9	93,9	0
H7C760	TR	A0A658QZE0	*Caballeronia concitans*	297	1388	88,9	93,3	0
H7C760	TR	A0A9N8QU21	*Paraburkholderia domus*	297	1388	88,9	93,6	0
H7C760	TR	A0A149Q121	*Paraburkholderia monticola*	297	1387	88,9	93,6	0
H7C760	TR	A0A158FLB8	*Caballeronia choica*	297	1377	88,9	92,3	0
H7C760	TR	A0A0L0MDC3	*Candidatus Burkholderia verschuerenii*	297	1376	88,9	92,6	0
H7C760	TR	A0A7Z2G2G3	*Paraburkholderia acidiphila*	297	1397	88,6	95,3	0
H7C760	TR	A0A372K162	*Paraburkholderia sp,* DHOC27	297	1386	88,6	93,9	0
H7C760	TR	Q13NT8	*Paraburkholderia xenovorans* (strain LB400)	297	1381	88,6	92,9	0
H7C760	TR	A0A1H7R4J4	*Paraburkholderia caballeronis*	297	1381	88,2	94,3	0
H7C760	TR	A0A1N6LGN1	*Paraburkholderia phenazinium*	297	1375	88,2	93,2	0
H7C760	TR	A0A160FI33	*Paraburkholderia phytofirmans* OLGA172	297	1374	88,2	94,3	0
H7C760	TR	A0A5B0GGY4	*Paraburkholderia panacisoli*	297	1363	88,2	92,2	0
H7C760	TR	Q144H6	*Paraburkholderia xenovorans* (strain LB400)	297	1373	87,9	92,6	0
H7C760	TR	A0A9X1UNL5	*Paraburkholderia tagetis*	297	1361	87,9	92,3	0
H7C760	TR	G4M6F1	*Candidatus Paraburkholderia kirkii* UZHbot1	298	1336	87,5	89,5	0
H7C761	TR	A0A1X7KTE4	*Paraburkholderia susongensis*	183	835	85,8	92,3	2,2E-112
H7C761	TR	A0A4R6F1X3	*Paraburkholderia sp,* BL10I2N1	183	820	85,6	92,2	4,3E-110
H7C761	TR	A0A1H0ZY16	*Paraburkholderia fungorum*	183	834	84,7	92,9	3,2E-112
H7C761	TR	A0A149Q0U2	*Paraburkholderia monticola*	183	828	84,7	91,8	2,6E-111
H7C761	TR	A0A1Q8J259	*Burkholderia sp,* SRS-W-2-2016	183	827	84,7	91,8	3,7E-111
H7C761	TR	A0A0L0MEG2	*Candidatus Burkholderia verschuerenii*	183	823	84,6	92,3	1,5E-110
H7C761	TR	A0A658QZ87	*Caballeronia concitans*	183	824	84,2	91,8	1,1E-110
H7C761	TR	A0A6J5FLU4	*Paraburkholderia fynbosensis*	183	823	84,2	92,3	1,5E-110
H7C761	TR	A0A1N7RMC4	*Paraburkholderia ribeironis*	183	821	84,2	91,3	3,1E-110
H7C761	TR	A0A0H3KCB1	*Burkholderia multivorans* (strain ATCC 17616 / 249)	183	825	84,1	92,3	7,5E-111
H7C761	TR	A0A1H7DK46	*Paraburkholderia diazotrophica*	183	828	83,6	92,3	2,6E-111
H7C761	TR	A0A9N8QYQ4	*Paraburkholderia domus*	183	824	83,6	92,3	1,1E-110
H7C761	TR	A0A6S7AX97	*Paraburkholderia ultramafica*	183	822	83,6	92,3	2,2E-110
H7C761	TR	A0A124P854	*Burkholderia singularis*	183	818	83,6	91,3	8,8E-110
H7C761	TR	A0A370MZF3	*Paraburkholderia lacunae*	183	816	83,6	91,8	1,8E-109
H7C761	TR	A0A2Z6EXS2	*Mycoavidus cysteinexigens*	183	811	83,6	90,7	1E-108
H7C772	TR	A0A1H7R578	*Paraburkholderia caballeronis*	342	1127	64,2	73,5	8,9E-152
H7C772	TR	A0A4U1IDQ2	*Trinickia terrae*	343	1104	63	73,7	2,9E-148
H7C772	TR	A0A1I3H1R7	*Paraburkholderia megapolitana*	344	1094	62,5	74,1	9,9E-147
H7C772	TR	A0A372K1X3	*Paraburkholderia sp,* DHOC27	346	1095	62,4	73,3	7,5E-147

H7C772	TR	A0A167VSV7	*Paraburkholderia phytofirmans* OLGA172	344	1105	62	73,4	2,1E-148
H7C772	TR	A0A1N6LGE2	*Paraburkholderia phenazinium*	344	1103	62	73,6	4,2E-148
H7C772	TR	A0A0L0MCZ9	*Candidatus Burkholderia verschuerenii*	344	1086	62	73,5	1,6E-145
H7C772	TR	A0A494XJE7	*Trinickia fusca*	343	1106	61,8	74,6	1,4E-148
H7C772	TR	A0A9X1UIH7	*Paraburkholderia tagetis*	333	1107	61,6	74,9	7,1E-149
H7C772	TR	A0A8T6ZBX1	*Paraburkholderia sacchari*	338	1103	61,6	73,8	3,4E-148
H7C772	TR	A0A2U3I6P5	*Caballeronia novacaledonica*	341	1100	61,4	75,6	1,1E-147
H7C777	TR	A0A118DM16	*Burkholderia singularis*	291	1175	80,4	86,6	1,1E-160
H7C777	TR	A0A9N8RSU3	*Paraburkholderia saeva*	281	921	74	82,2	3,6E-122
H7C777	TR	A0A4U1IDY3	*Trinickia terrae*	279	932	73,7	82,6	7,1E-124
H7C777	TR	A0A372K1P5	*Paraburkholderia sp,* DHOC27	279	929	72,2	80,2	2E-123
H7C777	TR	F2L7U3	*Burkholderia gladioli* (strain BSR3)	302	1020	71,4	79,9	6,6E-137
H7C777	TR	A0A158IYA3	*Caballeronia telluris*	269	893	71,3	83,1	4,2E-118
H7C777	TR	A0A149Q0R8	*Paraburkholderia monticola*	285	899	71,1	81,4	9,1E-119
H7C777	TR	A0A8A8D4B4	*Burkholderia seminalis*	285	1016	70,9	79,1	1,5E-136
H7C777	TR	B1FXN0	*Paraburkholderia graminis* (strain ATCC 700544 / DSM 17151 / LMG 18924 / NCIMB 13744 / C4D1M)	279	908	70,7	81,8	3,2E-120
H7C777	TR	A0A1H7R3N1	*Paraburkholderia caballeronis*	261	876	69,9	82	1,2E-115
H7C777	TR	A0A6J5BXI9	*Paraburkholderia sediminicola*	289	900	69,8	81,6	7,3E-119
H7C777	TR	A0A0Q5PVS6	*Burkholderia sp,* Leaf177	267	895	69,8	80,8	2,00E-118
H7C777	TR	A0A063BGW8	*Burkholderia sp,* lig30	285	977	69,7	79	1,30E-130
H7C777	TR	A0A1I3GZH5	*Paraburkholderia megapolitana*	256	895	69,6	80,6	1,30E-118
H7C777	TR	A0A1H0ZXY3	*Paraburkholderia fungorum*	259	894	69,6	82,1	2,10E-118
H7C777	TR	A0A0H3KL56	*Burkholderia multivorans* (strain ATCC 17616 / 249)	285	1016	69,4	78,9	1,50E-136
H7C777	TR	A0A658QZM0	*Caballeronia concitans*	271	878	69	81,6	8,70E-116
H7C777	TR	A0A1H7DRY3	*Paraburkholderia diazotrophica*	284	872	68,8	81,2	1,10E-114
H7C777	TR	A0A6G8NL88	*Caballeronia sp,* SBC1	269	887	68,7	80,3	3,50E-117
H7C777	TR	A0A158FJM6	*Caballeronia choica*	269	885	68,7	80,7	7,00E-117
H7C777	TR	A0A2S4MMF0	*Paraburkholderia eburnea*	249	865	68,6	80,2	3,80E-114
H7C777	TR	A0A0B6RIU2	*Burkholderia plantarii*	287	985	68,3	77,8	8,20E-132
H7C777	TR	A0A1N6LH59	*Paraburkholderia phenazinium*	298	925	68,3	79,6	1,60E-122
H7C777	TR	B2JFC9	*Paraburkholderia phymatum* (strain DSM 17167 / CIP 108236 / LMG 21445 / STM815)	283	878	68,3	82,9	1,30E-115
H7C777	TR	A0A7Z2G2F6	*Paraburkholderia acidiphila*	257	870	68,2	82,4	8,80E-115
H7C777	TR	A0A5B0GGV1	*Paraburkholderia panacisoli*	277	908	67,8	80,5	2,90E-120
H7C777	TR	A0A6J5FR50	*Paraburkholderia fynbosensis*	276	922	67,7	77,8	2,10E-122
H7C777	TR	A0A3N6NH16	*Paraburkholderia dinghuensis*	257	885	67,3	82,4	4,60E-117
H7C777	TR	A0A6J5FBH4	*Paraburkholderia caffeinitolerans*	257	856	66,7	82,1	1,20E-112
H7C777	TR	A0A069PH11	*Caballeronia glathei*	278	909	66,5	76,4	2,10E-120
H7C777	TR	A0A1X7KUU8	*Paraburkholderia susongensis*	281	909	66,4	76,3	2,40E-120
H7C777	TR	A0A2J6MUF0	*Burkholderia sp,* WAC0059	278	883	66,3	76,5	1,90E-116
H7C777	TR	A0A4R6EZN3	*Paraburkholderia sp,* BL10I2N1	278	893	66,2	74,6	5,80E-118
H7C777	TR	A0A0L0MEJ6	*Candidatus Burkholderia verschuerenii*	269	871	66,1	77,8	9,40E-115
H7C778	TR	A0A542ZIJ3	*Oryzihumus leptocrescens*	415	1018	51,2	65,5	6,3E-133
H7C778	TR	A0A4R8WJG3	*Cryobacterium sp,* MDB2-A-2	418	1001	49,2	66,8	2,6E-130
H7C778	TR	A0A0Q5E5H9	*Plantibacter sp,* Leaf314	422	878	47,1	65,7	1,1E-111
H7C778	TR	A0A7G6Z5H0	*Glaciihabitans sp,* INWT7	419	876	47,1	66,2	2,1E-111

H7C782	TR	A0A9N8MNH7	*Paraburkholderia domus*	307	1448	90,6	94,8	0
H7C782	TR	A0A7W6N9E6	*Microvirga flocculans*	312	817	52	70,2	1,2E-105
H7C782	TR	A0A542ZI24	*Oryzihumus leptocrescens*	302	822	51,2	69,1	1,5E-106
H7C782	TR	A0A9N8MNI8	*Paraburkholderia domus*	310	779	49,2	68,7	6,7E-100
H7C782	TR	A0A0Q4Y0F7	*Pseudorhodoferax sp,* Leaf267	297	773	49,2	68,8	3,5E-99
Q63RJ0	TR	A0A9N8MPL5	*Paraburkholderia domus*	289	1131	78,3	87,4	3,3E-154
Q63RJ0	TR	A0A4R2RJM4	*Heliophilum fasciatum*	276	869	62,8	79,6	1,5E-114
Q63RJ0	TR	F6B5J8	*Desulfotomaculum nigrificans* (strain DSM 14880 / VKM B-2319 / CO-1-SRB)	294	880	61,7	79,9	6E-116
Q63RJ0	TR	A0A080M8K4	*Candidatus Accumulibacter cognatus*	263	811	61,7	76,9	6,3E-106
Q63RJ5	TR	A0A9N8QU26	*Paraburkholderia domus*	574	2277	73,9	84,1	0
Q63RJ5	TR	A0A7W8JPZ5	*Rhodanobacter sp,* ANJX3	623	1162	48,9	61,1	2,7E-149
Q63RJ5	TR	A0A542ZHY7	*Oryzihumus leptocrescens*	583	1303	47,1	61,5	4,3E-171
Q63RJ5	TR	A0A0Q4XY64	*Pseudorhodoferax sp,* Leaf267	345	554	45,5	64,8	2,6E-62
Q63RJ6	TR	A0A9N8QYR0	*Paraburkholderia domus*	613	2271	70	81,1	0
Q63RJ6	TR	A0A6M4H3V8	*Usitatibacter palustris*	610	1296	47	60,2	4,5E-169
Q63RJ6	TR	A0A7X1E348	*Puniceicoccus vermicola*	550	336	44,7	58,4	9,2E-30
Q63RJ6	TR	A0A7X0JPW3	*Pseudoteredinibacter isoporae*	594	1228	44,5	62,1	4,7E-159
Q63RJ6	TR	A0A6M4GTY7	*Usitatibacter rugosus*	611	1207	44,4	58,8	1,2E-155
Q63RJ7	TR	A0A9N8QYR6	*Paraburkholderia domus*	281	1006	68,1	78,4	2,6E-135
Q63RJ7	TR	A0A4R6F0Y0	*Paraburkholderia sp,* BL10I2N1	281	992	65,8	77,1	3,5E-133
Q63RJ7	TR	A0A1N6LGN3	*Paraburkholderia phenazinium*	281	972	65,5	76	3,8E-130
Q63RJ7	TR	A0A2N7VFD2	*Trinickia dabaoshanensis*	281	951	65,3	78,4	6E-127
Q63RJ7	TR	A0A1I3H1R0	*Paraburkholderia megapolitana*	280	984	65,2	78,8	5,5E-132
Q63RJ7	TR	Q144M4	*Paraburkholderia xenovorans* (strain LB400)	281	973	64,4	74,5	2,7E-130
Q63RJ7	TR	A0A419Z2C3	*Paraburkholderia sp,* BL23I1N1	281	940	64,2	76,5	2,8E-125
Q63RJ7	TR	A0A372K147	*Paraburkholderia sp,* DHOC27	281	938	64,1	75,5	5,7E-125
Q63RJ7	TR	B2JF99	*Paraburkholderia phymatum* (strain DSM 17167 / CIP 108236 / LMG 21445 / STM815)	281	972	63,7	79,1	3,8E-130
Q63RJ7	TR	A0A4U1IDP6	*Trinickia terrae*	281	937	63,3	75,6	8E-125
Q63RJ7	TR	A0A160FH85	*Paraburkholderia phytofirmans* OLGA172	281	931	63,3	74,2	6,6E-124
Q63RJ7	TR	A0A8T6ZAH2	*Paraburkholderia sacchari*	278	894	62,9	73,9	2,5E-118
Q63RJ7	TR	A0A0H3KHD0	*Burkholderia multivorans* (strain ATCC 17616 / 249)	288	904	62,2	76	1,1E-119
Q63RJ7	TR	A0A8A8D436	*Burkholderia seminalis*	281	874	62	74,1	3E-115
Q63RJ7	TR	A0A1H7DL50	*Paraburkholderia diazotrophica*	281	914	61,8	72,7	2,5E-121
Q63RJ7	TR	A0A494XJE9	*Trinickia fusca*	291	908	61,8	74,6	2,9E-120
Q63RJ7	TR	A0A1N7S4N9	*Paraburkholderia ribeironis*	280	873	61,8	72,1	4,2E-115
Q63RJ7	TR	W2TYW9	*Necator americanus*	298	901	61,6	73,1	4,3E-119
Q63RJ7	TR	A0A7Z2GJG2	*Paraburkholderia acidisoli*	281	889	61,6	73,9	1,6E-117
Q63RJ7	TR	A0A1X7CIT3	*Trinickia caryophylli*	305	883	61,6	75,3	3E-116
Q63RJ7	TR	A0A1Q8J299	*Burkholderia sp,* SRS-W-2-2016	282	914	61,5	76	2,6E-121
Q63RJ7	TR	A0A4R0XKT5	*Paraburkholderia steynii*	270	880	61,1	75,2	2,5E-116
Q63RJ7	TR	A0AAJ2E2N0	*Caballeronia sp,* LZ002	274	893	61	76	3,1E-118
Q63RJ8	TR	A0A124P852	*Burkholderia singularis*	319	1051	65,1	76,7	7,9E-141
Q63RJ8	TR	B2JF98	*Paraburkholderia phymatum* (strain DSM 17167 / CIP 108236 / LMG 21445 / STM815)	318	1038	64	75,4	7,3E-139
Q63RJ8	TR	A0A1I3H1I7	*Paraburkholderia megapolitana*	318	1011	63,4	76,3	9,2E-135
Q63RJ8	TR	Q144M3	*Paraburkholderia xenovorans* (strain LB400)	318	1011	63,1	75,4	9,2E-135

Q63RJ8	TR	A0A1Q8J228	*Burkholderia sp,* SRS-W-2-2016	320	1035	62,9	75,4	2,2E-138
Q63RJ8	TR	A0A0B6RSX9	*Burkholderia plantarii*	318	993	62,7	75,8	5E-132
Q63RJ8	TR	A0A940NHE6	*Paraburkholderia sp,* LEh10	321	1023	62,5	76,3	1,5E-136
Q63RJ8	TR	A0A4V5PL19	*Trinickia terrae*	318	1014	61,8	77	3,2E-135
Q63RJ8	TR	A0A4R6EZJ0	*Paraburkholderia sp,* BL10I2N1	318	1008	61,8	74,1	2,6E-134
Q63RJ8	TR	A0A1H7R5B5	*Paraburkholderia caballeronis*	330	1005	61,6	73,6	1,1E-133
Q63RJ8	TR	A0A7Z2JF58	*Paraburkholderia acidisoli*	325	987	61,4	75,3	5,2E-131
Q63RJ8	TR	A0A2J6MLU4	*Burkholderia sp,* WAC0059	325	988	61	73,3	3,7E-131
Q63RJ8	TR	A0A9X1UIP1	*Paraburkholderia tagetis*	318	975	60,9	72,9	2,7E-129
Q63RJ8	TR	A0A9N8MPG3	*Paraburkholderia domus*	318	984	60,6	73,5	1,2E-130
Q63RJ8	TR	A0A1H7DKL7	*Paraburkholderia diazotrophica*	318	972	60,6	73,5	7,7E-129
Q63RJ8	TR	A0A3R7G6K9	*Paraburkholderia sp,* BL23I1N1	320	984	60,3	73,2	1,2E-130
Q63RJ8	TR	A0A160FH90	*Paraburkholderia phytofirmans* OLGA172	318	973	60,3	74,8	5,4E-129
Q63RJ8	TR	A0A1X7CID8	*Trinickia caryophylli*	321	979	60	73,3	7,4E-130
Q63RJ8	TR	W2TXR6	*Necator americanus*	241	741	60	76,7	6,3E-95
Q63RJ8	TR	A0A9P6TLA5	*Apophysomyces sp,* BC1034	581	962	59,8	71	1,2E-123
Q63RK0	TR	A0A103DX10	*Burkholderia singularis*	632	2918	90	94,3	0
Q63RK0	TR	A0A0H3KCS7	*Burkholderia multivorans* (strain ATCC 17616 / 249)	628	2821	88	93,8	0
Q63RK0	TR	F2L7W1	*Burkholderia gladioli (strain BSR3)*	624	2743	86,7	92,4	0
Q63RK0	TR	W2U0I2	*Necator americanus*	781	2137	86,5	93,1	0
Q63RK0	TR	A0A0B6RZ46	*Burkholderia plantarii*	627	2744	86,4	91,6	0
Q63RK0	TR	A0A4U1IDN7	*Trinickia terrae*	630	2725	84,2	91,6	0
Q63RK0	TR	A0A1N6LGC1	*Paraburkholderia phenazinium*	627	2695	83,8	92,2	0
Q63RK0	TR	A0A069PW88	*Caballeronia glathei*	635	2676	83,1	91	0

Result 5 : **Multiple sequence alignment of the 24 Capsular Polysaccharide (CPS) biosynthesis proteins from *Burkholderia pseudomallei*.** Top five homologous sequences for each protein were identified using BLAST searches against the NCBI non-redundant (nr) protein database, excluding *Burkholderia pseudomallei*. Uniprot ID, unique identifier of the protein in the UniProt database ; DB, source database from which the sequence information was retrieved ; Accession, accession number of the matched sequence from BLAST results ; Organism, species from which the matched sequence originates ; Length, total number of amino acids in the matched protein ; Score (Bits), alignment score reflecting the quality of the BLAST match : higher scores indicate better matches ; Identities (%), percentage of amino acids that are identical between the query and subject sequences ; Positives (%), percentage of amino acids that are either identical or similar (conservative substitutions ; E(), expectation value indicating the likelihood of the match occurring by chance; lower values denote more significant matches.

Uniprot ID	DB	Accession	Organism	Length	Score(Bits)	Identities(%)	Positives(%)	E()
Q63R84	TR	A0A8A8D435	*Burkholderia seminalis*	2542	11650	90,1	93,7	0
Q63R86	TR	A0A8A8D442	*Burkholderia seminalis*	442	2018	89	93,6	0
H7C736	TR	A0A249DXS9	*Candidatus Hamiltonella defensa (Bemisia tabaci)*	335	1496	84,1	92,2	0
H7C781	TR	A0A103E720	*Burkholderia singularis*	476	2030	81,4	90.5	0
Q63R85	TR	A0A0B6RSM8	*Burkholderia plantarii*	307	1264	80,2	88,1	1,1E-173
H7C745	TR	A0A4U0Q7X6	*Chitiniphilus eburneus*	341	1414	79,1	87,6	0
H7C767	TR	A0A6S7CVJ6	*Paraburkholderia ultramafica*	388	1515	78	87,4	0
BPSL2795	TR	A0A4U0Q8V1	*Chitiniphilus eburneus*	176	729	77,9	90.7	4,40E-96
Q63R82	TR	A0A6S7BHZ0	*Paraburkholderia ultramafica*	261	975	77,3	83,9	3,8E-131
Q63R65	TR	A0A6S7B649	*Paraburkholderia ultramafica*	414	1416	72,1	81,6	0
Q63R81	TR	A0A6S7BK00	*Paraburkholderia ultramafica*	402	1522	70,2	81	0
Q63R69	TR	A0A841L6E8	*Polymorphobacter multimanifer*	217	781	69,9	79,2	4,9E-103
Q63R83	TR	W2TYX9	*Necator americanus*	315	1142	69,7	80,3	1E-151
Q63R68	TR	A0A843YP76	*Glaciimonas soli*	263	916	68,6	80	3,6E-122
Q63R80	TR	A0A346R2C5	*Breoghania sp,* L-A4	190	501	65,1	73,8	2,4E-61
Q63R64	TR	A0A6S7BA81	*Pararobbsia alpina*	680	2118	64,9	75	0
Q63R79	TR	A0A1N6UTQ3	*Aromatoleum tolulyticum*	228	729	63,6	76	9,3E-95
Q63R74	TR	J3KVS5	*Oryza brachyantha*	606	92	57,1	68,6	4,2
H7C733	TR	A0A512L6I1	*Sulfuriferula plumbiphila*	385	978	53	73,6	8,9E-128
Q63R70	TR	A0A410UW15	*Janthinobacterium sp,* 17J80-10	506	1117	45,4	63,1	4E-145
H7C744	TR	A0A3F2V4Y5	*Ketobacter sp,*	299	579	44,1	60,6	3,1E-70
H7C730	TR	A0A1X6NPD2	*Porphyra umbilicalis*	420	91	43,8	53,1	4,9
Q63R73	TR	H2CAQ6	*Leptonema illini* DSM 21528	369	153	39,8	55,1	0,00000031
Q63R71	TR	A0A7V8QFF2	*Ectothiorhodospiraceae bacterium* WFHF3C12	301	439	36,4	55,9	2,6E-47
Q63R86	TR	A0A103DX69	*Burkholderia singularis*	440	1969	87,3	93,2	0
Q63R86	TR	F2LAL5	*Burkholderia gladioli* (strain BSR3)	438	1886	84,5	90,2	0
Q63R86	TR	A0A1X7LRP6	*Paraburkholderia susongensis*	440	1897	83,5	91,8	0
Q63R86	TR	A0A0B6RVU9	*Burkholderia plantarii*	439	1866	83,1	89,3	0
Q63R86	TR	A0A6J5GUM2	*Paraburkholderia fynbosensis*	444	1884	83	89,6	0
Q63R86	TR	A0A5B0GZB9	*Paraburkholderia panacisoli*	437	1873	82,5	90,4	0
Q63R86	TR	A0A1Q8IPS1	*Burkholderia sp,* SRS-W-2-2016	443	1865	82,1	89,8	0
Q63R86	TR	A0A6S7B6R3	*Paraburkholderia ultramafica*	445	1832	80,9	87,9	0

Q63R86	TR	A0A1H7HA65	*Paraburkholderia caballeronis*	445	1815	80,5	87,6	0
Q63R85	TR	F2LAL4	*Burkholderia gladioli* (strain BSR3)	306	1247	80,1	88	4E-171
Q63R86	TR	A0A3N6NLJ0	*Paraburkholderia dinghuensis*	446	1817	79,8	88,7	0
Q63R86	TR	A0A6S7AYU5	*Pararobbsia alpina*	445	1803	79,7	86,9	0
H7C781	TR	A0A6S7CVN2	*Paraburkholderia ultramafica*	476	1974	78,9	88.2	0
Q63R86	TR	A0A2S4MMI1	*Paraburkholderia eburnea*	446	1785	78,7	87	0
H7C736	TR	A0A917C843	*Terasakiella brassicae*	336	1391	78,6	87,2	0
H7C781	TR	A0AAD5UNU7	*Physisporinus lineatus*	398	1623	78,5	89.0	0
Q63R85	TR	A0A118DM41	*Burkholderia singularis*	302	1244	78,5	86,1	1E-170
H7C736	TR	A0A2T6BNV5	*Litoreibacter ponti*	336	1418	77,7	89,6	0
H7C767	TR	A0AAD5YBQ0	*Physisporinus lineatus*	197	825	77,2	92,4	2,5E-107
H7C781	TR	A0A370N174	*Paraburkholderia lacunae*	476	1899	76,9	87.1	0
H7C736	TR	A0A1T4KIN8	*Enhydrobacter aerosaccus*	339	1392	76,9	88,7	0
BPSL2795	TR	A0A3F2V519	*Ketobacter sp.*	176	697	76,7	87.8	3,30E-91
H7C745	TR	A0A1N6UTL5	*Aromatoleum tolulyticum*	342	1389	76,5	87,7	0
H7C781	TR	F2L9A8	*Burkholderia gladioli* (strain BSR3)	474	1892	76,4	86.2	0
H7C781	TR	A0A6S7CKP7	*Pararobbsia alpina*	392	1504	76,1	85.7	0
H7C781	TR	A0A160FVT4	*Paraburkholderia phytofirmans* OLGA172	477	1924	75,9	87.8	0
H7C781	TR	A0A494XAZ3	*Pararobbsia silviterrae*	476	1872	75,8	85.4	0
H7C781	TR	A0A1D9GV93	*Cupriavidus sp.* USMAA2-4	475	1913	75,7	86.5	0
BPSL2795	TR	A0A4R5W273	*Sapientia aquatica*	198	752	75,3	85.1	2,90E-99
H7C745	TR	A0A5Q0BGT1	*Candidatus Methylospira mobilis*	344	1359	75,3	86,5	0
H7C745	TR	A0A4R5W2F7	*Sapientia aquatica*	341	1309	75,3	83,8	2,6E-179
Q63R86	TR	U3GDT5	*Ralstonia sp,* 5_2_56FAA	453	1693	74,8	82,5	0
Q63R82	TR	A0A6S7B7J3	*Pararobbsia alpina*	261	959	74,4	82,9	1E-128
Q63R85	TR	A0A2S4MMA7	*Paraburkholderia eburnea*	312	1179	74,4	81,6	1,1E-160
Q63R86	TR	A0A2U3I5Z6	*Caballeronia novacaledonica*	442	1659	73,8	82,8	0
Q63R82	TR	A0A1H7H9V0	*Paraburkholderia caballeronis*	261	921	72,9	81,6	6,3E-123
Q63R85	TR	A0A6S7AYN2	*Pararobbsia alpina*	311	1166	72,5	83,1	1E-158
Q63R82	TR	A0A0B6RYV7	*Burkholderia plantarii*	263	938	72,4	83,1	1,8E-125
BPSL2795	TR	A0A3M6QMK5	*Corticibacter populi*	194	690	72,2	81.8	7E-90
Q63R82	TR	A0A2S4MML7	*Paraburkholderia eburnea*	261	950	71,9	82	2,4E-127
Q63R84	TR	A0A0B6RP87	*Burkholderia plantarii*	2533	9095	71,8	80,5	0
Q63R65	TR	A0A6S7AZL3	*Pararobbsia alpina*	383	1433	71,5	80,1	0
Q63R82	TR	A0A494XGN4	*Pararobbsia silviterrae*	261	900	71,3	82,3	9,9E-120
Q63R85	TR	A0A3N6QBV3	*Paraburkholderia dinghuensis*	309	1135	71,2	80,7	5E-154
Q63R85	TR	A0A2U3I5Y5	*Caballeronia novacaledonica*	307	1119	71,1	81,2	1,3E-151
Q63R84	TR	F2LAL3	*Burkholderia gladioli* (strain BSR3)	2550	8923	70,8	80,1	0
Q63R85	TR	A0A1Q8IPT5	*Burkholderia sp,* SRS-W-2-2016	317	1119	70,8	81,3	1,8E-151
Q63R85	TR	A0A6J5GVN2	*Paraburkholderia fynbosensis*	317	1113	70,8	81,3	1,5E-150
H7C767	TR	T2N073	*Ralstonia sp,* 5_2_56FAA	388	1352	69,9	81,9	0
Q63R82	TR	A0A118DM27	*Burkholderia singularis*	261	924	69,9	79,9	2,2E-123
Q63R85	TR	A0A6S7CGA8	*Paraburkholderia ultramafica*	304	1100	69,8	80,3	8,9E-149
Q63R85	TR	A0A1X7LTB1	*Paraburkholderia susongensis*	317	1098	69,8	79,7	2,9E-148
Q63R82	TR	W2TYE9	*Necator americanus*	616	834	69,6	80,6	6E-105

H7C767	TR	A0A103E773	*Burkholderia singularis*	397	1377	69,5	81,9	0
Q63R81	TR	A0A103DXA1	*Burkholderia singularis*	395	1482	69,3	81,1	0
Q63R69	TR	A0A345P3V7	*Aquirhabdus parva*	217	785	68,7	81,3	1,2E-103
Q63R81	TR	W2TYE9	*Necator americanus*	616	1491	68,7	80,5	0
Q63R81	TR	A0A6S7AZ08	*Pararobbsia alpina*	402	1472	68,5	80	0
Q63R82	TR	U3GDT1	*Ralstonia sp,* 5_2_56FAA	261	875	68,4	78,5	6,3E-116
Q63R85	TR	A0A5B0GZ96	*Paraburkholderia panacisoli*	304	1103	68,3	80,5	3,1E-149
Q63R83	TR	A0A2S4MM84	*Paraburkholderia eburnea*	514	1765	68,2	77,7	0
H7C767	TR	A0A0B6RIK0	*Burkholderia plantarii*	394	1366	68,1	81,1	0
Q63R84	TR	A0A1H7HBM1	*Paraburkholderia caballeronis*	2539	8506	68,1	77	0
Q63R65	TR	A0A8A8D3J2	*Burkholderia seminalis*	383	1398	67,9	80,9	0
H7C767	TR	A0A494XB25	*Pararobbsia silviterrae*	391	1349	67,9	81,7	0
Q63R84	TR	A0A6J5GV77	*Paraburkholderia fynbosensis*	2552	8367	67,7	76,6	0
Q63R84	TR	A0A5B0GZ47	*Paraburkholderia panacisoli*	2540	8359	67,7	76,4	0
Q63R85	TR	U3GDT4	*Ralstonia sp,* 5_2_56FAA	297	1028	67,7	79,3	6,2E-138
Q63R84	TR	A0A118DM76	*Burkholderia singularis*	2569	8436	67,6	76,4	0
Q63R65	TR	A0AAD5Y943	*Physisporinus lineatus*	280	1009	67,5	78,6	4,9E-134
Q63R69	TR	A0A2S4MM95	*Paraburkholderia eburnea*	216	767	67,5	81,6	6,5E-101
Q63R81	TR	U3GDT0	*Ralstonia sp,* 5_2_56FAA	402	1407	67,5	76,2	0
Q63R84	TR	A0A1Q8IPX8	*Burkholderia sp,* SRS-W-2-2016	2564	8340	67,4	76,3	0
Q63R65	TR	U3GDS1	*Ralstonia sp,* 5_2_56FAA	261	933	67,3	78,5	8,6E-123
Q63R69	TR	A0A2S5SXG9	*Caldimonas caldifontis*	217	782	67,3	82,2	3,5E-103
Q63R84	TR	A0A1X7LRM4	*Paraburkholderia susongensis*	2551	8319	67,2	76,5	0
Q63R68	TR	A0A2S5SXI4	*Caldimonas caldifontis*	265	922	66,7	80	4,7E-123
Q63R68	TR	A0A345PB42	*Aquirhabdus parva*	242	880	66,5	80,6	5,2E-117
Q63R84	TR	A0A6S7B698	*Paraburkholderia ultramafica*	2559	8266	66,1	75,9	0
Q63R84	TR	A0A3N6NM64	*Paraburkholderia dinghuensis*	2538	8067	66,1	74,6	0
Q63R84	TR	A0A2S4MMB1	*Paraburkholderia eburnea*	2541	8153	65,6	75,3	0
Q63R68	TR	A0A6N1X5X6	*Comamonas antarctica*	268	900	64,5	80,7	1,2E-119
Q63R84	TR	A0A6S7B7U8	*Pararobbsia alpina*	2566	8082	64,2	74,9	0
Q63R80	TR	A0A4R5W4L5	*Sapientia aquatica*	185	501	63,9	74,8	2,1E-61
Q63R64	TR	A0A6S7BHX4	*Paraburkholderia ultramafica*	685	2097	63,3	75,3	0
Q63R84	TR	A0A494XAA1	*Pararobbsia silviterrae*	2604	7745	63,2	71,7	0
Q63R83	TR	A0A1H7HA06	*Paraburkholderia caballeronis*	512	1576	63,1	72,6	0
Q63R79	TR	A0A4V3AUV3	*Sapientia aquatica*	228	746	62,6	78,9	2,4E-97
Q63R80	TR	A0A0F3ILF6	*Methylocucumis oryzae*	179	519	62,1	75,8	3,2E-64
Q63R80	TR	A0A2N5XS65	*Cohaesibacter celericrescens*	183	476	61,8	72,4	1,2E-57
Q63R83	TR	A0A6S7CVM0	*Paraburkholderia ultramafica*	510	1576	61,5	75,1	0
Q63R83	TR	F2LAL2	*Burkholderia gladioli* (strain BSR3)	513	1483	61,4	72,5	0
Q63R83	TR	A0A118DM75	*Burkholderia singularis*	511	1586	60,8	72,7	0
Q63R84	TR	A0A2U3I603	*Caballeronia novacaledonica*	2218	6443	60,6	70,9	0
Q63R79	TR	A0A4U0Q7T2	*Chitiniphilus eburneus*	229	689	60,4	72,9	1,2E-88
Q63R83	TR	A0A6S7B1B8	*Pararobbsia alpina*	511	1500	60,4	73,2	0
Q63R79	TR	A0A3F2V549	*Ketobacter sp,*	229	692	60,3	72,5	4,1E-89
Q63R83	TR	A0A0B6RIK8	*Burkholderia plantarii*	509	1460	59,6	68,9	0
Q63R84	TR	U3GDT3	*Ralstonia sp,* 5_2_56FAA	2556	7188	59,3	70,9	0

Q63R64	TR	A0A2U3I665	*Caballeronia novacaledonica*	673	1856	58,8	69,4	0
Q63R83	TR	A0A5B0GZ44	*Paraburkholderia panacisoli*	511	1425	58,5	69,5	0
Q63R64	TR	A0A2S4MMT1	*Paraburkholderia eburnea*	697	1825	57,3	68,1	0
Q63R64	TR	A0A0B6RVT0	*Burkholderia plantarii*	669	1707	56,5	67,8	0
Q63R83	TR	A0A3N6P7N2	*Paraburkholderia dinghuensis*	510	1363	56,2	67,4	0
Q63R64	TR	A0A8A8D3W6	*Burkholderia seminalis*	667	1741	55,8	66,5	0
Q63R83	TR	A0A1Q8IPX5	*Burkholderia sp,* SRS-W-2-2016	509	1352	55,8	67,7	1,4E-180
Q63R83	TR	A0A6J5GWE7	*Paraburkholderia fynbosensis*	475	1264	55,8	66	9,3E-168
Q63R83	TR	A0A1X7LRQ0	*Paraburkholderia susongensis*	475	1290	55,5	67,1	1,1E-171
Q63R64	TR	F2L9A9	*Burkholderia gladioli* (strain BSR3)	671	1641	55,2	68	0
Q63R64	TR	A0A103E724	*Burkholderia singularis*	672	1750	54,6	67,6	0
Q63R64	TR	U3GDS0	*Ralstonia sp,* 5_2_56FAA	671	1713	54,3	66,4	0
Q63R83	TR	A0A2U3I623	*Caballeronia novacaledonica*	516	1362	54,3	69,7	0
H7C733	TR	A0A410UW45	*Janthinobacterium sp,* 17J80-10	383	978	52,4	75,5	8,3E-128
H7C733	TR	A0A843YTZ0	*Glaciimonas soli*	384	980	51,9	74,2	4,3E-128
H7C733	TR	A0A1W7MBL7	*Novosphingobium sp,* MD-1	206	528	51,9	68,4	2E-62
H7C733	TR	A0A6N1XAM1	*Comamonas antarctica*	384	927	50,1	73	4,5E-120
Q63R70	TR	A0A554X5T1	*Tepidimonas charontis*	515	1099	44,5	62,5	2,8E-142
Q63R70	TR	A0A179BDL6	*Acidithiobacillus ferrooxidans*	516	1044	44,5	58,4	5,5E-134
Q63R70	TR	A0A848HDB9	*Ramlibacter agri*	503	1065	44,4	61,2	2,5E-137
H7C744	TR	L8LFY7	*Gloeocapsa sp,* PCC 73106	287	509	41,5	58,5	7,4E-60
H7C730	TR	A0A923ESH4	*Pseudonocardia sp,* C8	257	89	39,4	56,3	6,3
H7C744	TR	A0A3A9T2J2	*Butyrivibrio sp,* XB500-5	318	459	38,3	51,4	5,5E-52
Q63R74	TR	V4QFV1	*Asticcacaulis sp,* YBE204	274	105	38,1	58,7	0,07
Q63R74	TR	A0A2V1H1D8	*Pelagibaculum spongiae*	483	118	37,5	60,9	0,0024
H7C744	TR	A0A1T4VC02	*Succinivibrio dextrinosolvens* DSM 3072	287	448	37,5	55	1E-50
Q63R71	TR	A0A7X0NG86	*Thalassotalea piscium*	460	411	36	54,1	1,1E-41
Q63R73	TR	A0A3M8FZV9	*Phycisphaera sp,*	422	147	34,7	47,9	0,0000021
Q63R74	TR	V4P1E6	*Asticcacaulis sp,* YBE204	278	110	34,3	58,6	0,02
Q63R74	TR	A0A1V0RSX1	*Roseovarius mucosus*	290	95	33,9	51,6	1,2
Q63R71	TR	A0A8A7KGD6	*Iocasia fonsfrigidae*	169	141	33,6	55,2	0,00000079
Q63R73	TR	A0A6G7Z7F9	*Sanguibacter sp,* HDW7	395	139	33,6	46	0,000018
Q63R71	TR	A0A2S6NBV3	*Rhodopila globiformis*	264	138	32,9	46,9	0,0000071
Q63R73	TR	A0A5B2VE06	*Salinarimonas soli*	359	144	32,9	44,3	0,0000038
Q63R71	TR	A0A3N6NKH2	*Okeania hirsuta*	259	142	32,7	48	0,0000021
H7C730	TR	A0A8S3XFL7	*Parnassius apollo*	142	90	31,4	51,2	1,9
H7C730	TR	A0A1L9B1E2	*Cystobacter ferrugineus*	739	101	29,4	47,1	0,35

Result 6 : **DNA concentration from enriched soil and water extracted samples measured using multiskan sky (Thermoscientific , Singapore).**

Samples	Concentration
Soil 1	10,6 µg/mL
Soil 2	2,4 µg/mL
Soil 3	1,6 µg/mL
Soil 4	0,1 µg/mL
Soil 5	0,1 µg/mL
Soil 6	2,4 µg/mL
Soil 7	2,3 µg/mL
Soil 8	3,5 µg/mL
Soil 9	0,6 µg/mL
Soil 10	0,1 µg/mL
Soil 11	5,7 µg/mL
Soil 12	0,1 µg/mL
Soil 13	1,6 µg/mL
Soil 14	10,2 µg/mL
Soil 15	4,7 µg/mL
Soil 16	13,8 µg/mL
Soil 17	14,7 µg/mL
Soil 18	13,4 µg/mL
Soil 19	2,6 µg/mL
Soil 20	4,5 µg/mL
Soil 21	6,5 µg/mL
Soil 22	14,9 µg/mL
Soil 23	2,7 µg/mL
Soil 24	3,7 µg/mL
Soil 25	4,6 µg/mL
Soil 26	2,1 µg/mL
Soil 27	2 µg/mL
Soil 28	9,8 µg/mL
Soil 29	11,1 µg/mL
Soil 30	3,1 µg/mL
Soil 31	8,7 µg/mL
Soil 32	10,5 µg/mL
Soil 33	11,5 µg/mL
Soil 34	7 µg/mL
Soil 35	7,7 µg/mL
Soil 36	17,3 µg/mL
Soil 37	13,6 µg/mL
Soil 38	9,5 µg/mL
Soil 39	4,2 µg/mL
Soil 40	7,1 µg/mL
Soil 41	0,1 µg/mL
Soil 42	1 µg/mL
Soil 43	11,2 µg/mL

Soil 44	15,8 µg/mL
Soil 45	5,1 µg/mL
Soil 46	9,2 µg/mL
Soil 47	5,5 µg/mL
Soil 48	5 µg/mL
Soil 49	7,5 µg/mL
Soil 50	7,7 µg/mL
Soil 51	10,6 µg/mL
Soil 52	4,4 µg/mL
Soil 53	3,7 µg/mL
Soil 54	7,2 µg/mL
Soil 55	7,1 µg/mL
Soil 56	0,1 µg/mL
Soil 57	6,9 µg/mL
Soil 58	16,8 µg/mL
Soil 59	9 µg/mL
Soil 60	3,7 µg/mL
Soil 61	0,1 µg/mL
Soil 62	0,1 µg/mL
Soil 63	4,2 µg/mL
Soil 64	3,1 µg/mL
Soil 65	8 µg/mL
Soil 66	0,5 µg/mL
Soil 67	1,3 µg/mL
Soil 68	0,4 µg/mL
Soil 69	0,1 µg/mL
Water 1	93,3 µg/mL
Water 2	266 µg/mL
Water 3	49,9 µg/mL
Water 4	172 µg/mL

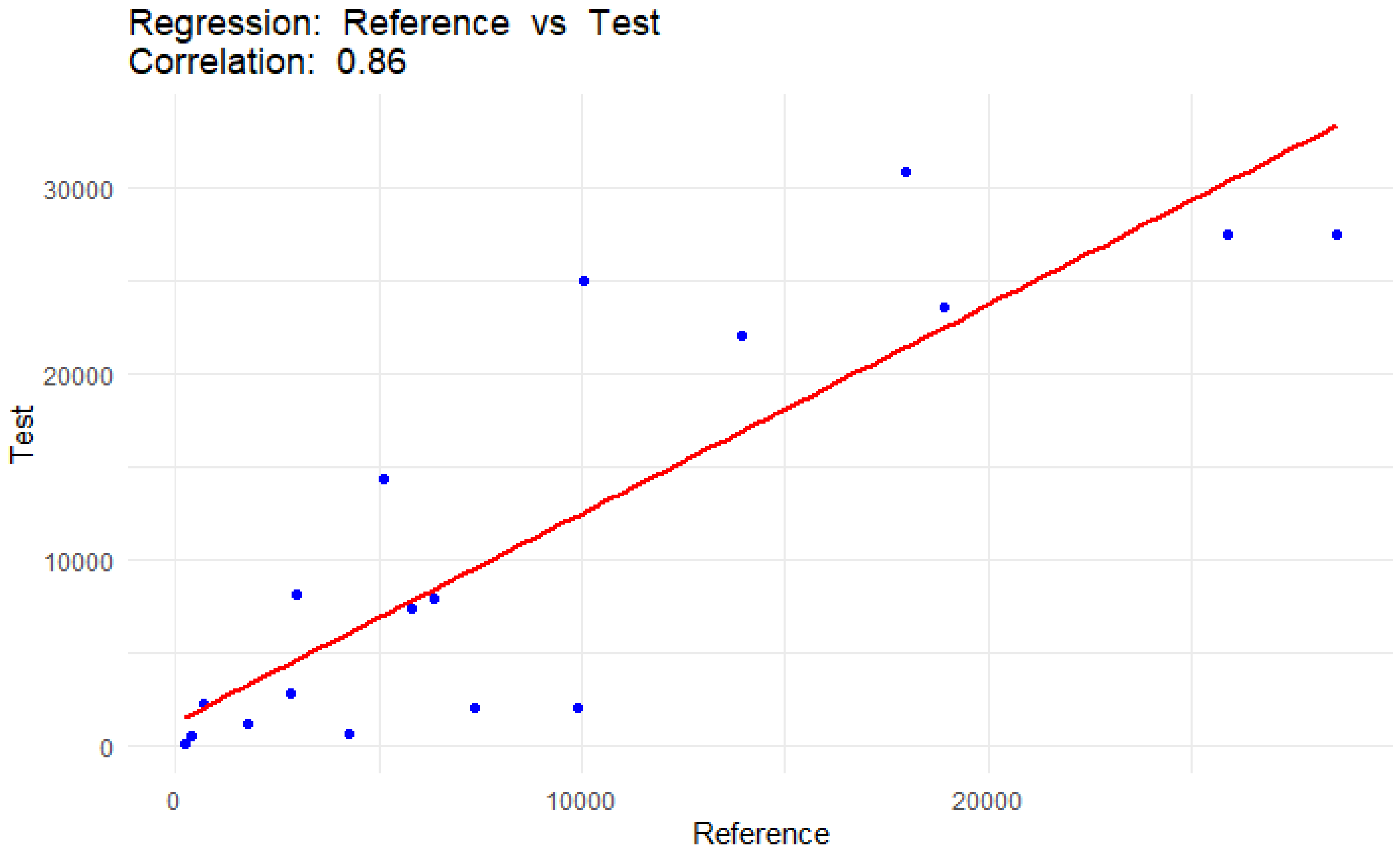

Fig A : Correlation of median fluorescence intensities from reference samples tested at Northern Arizona University (Reference) and the Immunology of Infectious Diseases Unit, Pasteur Institute of Madagascar (Test). Correlation indicated by the Pearson correlation coefficient.

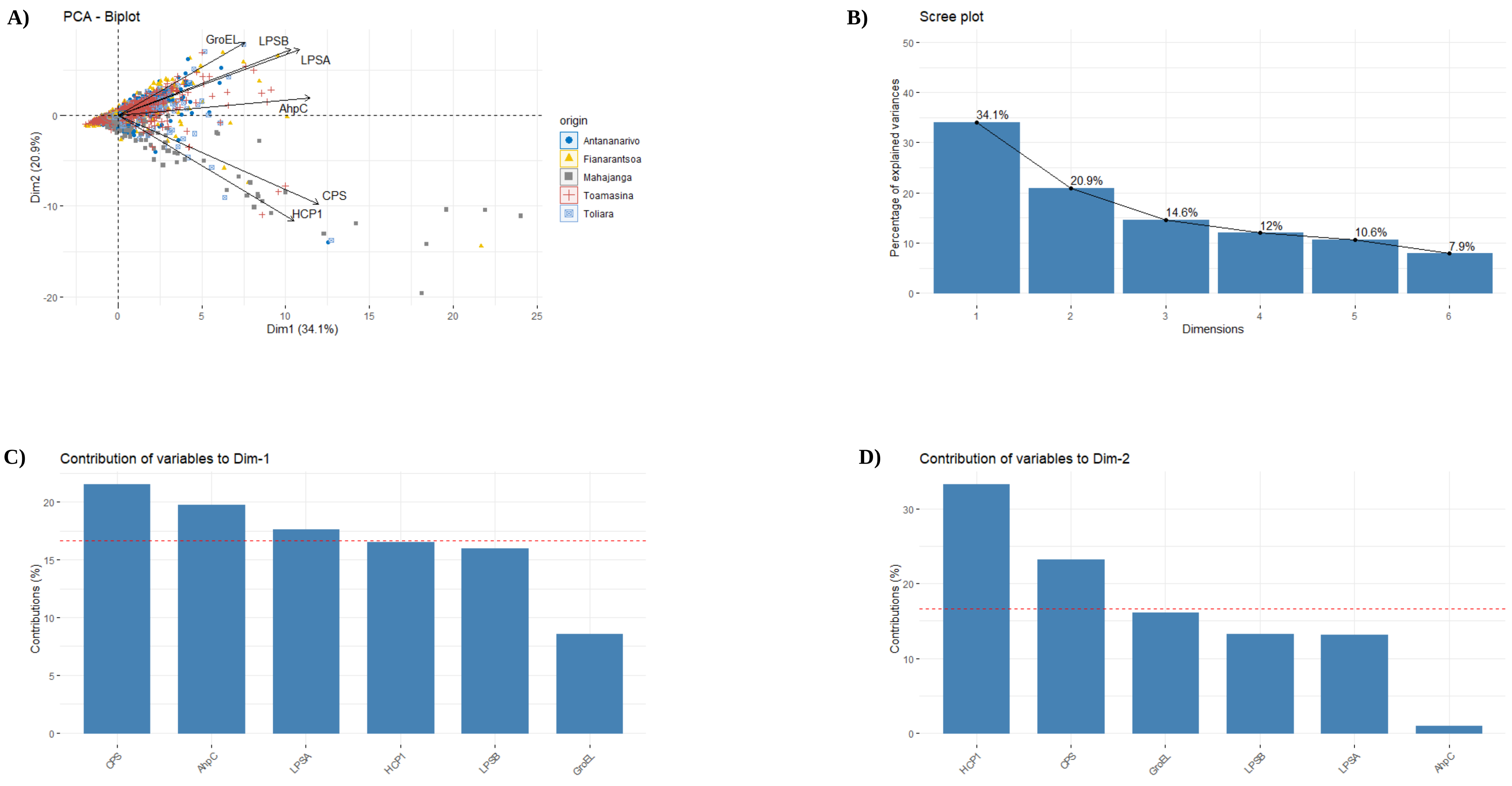

Fig B : Principal component analysis of reactivity to the six antigens. A**)** PCA Variable Plot Showing the Contribution of Six *B. pseudomallei* Antigens (GroEL, AhpC, HCP1, CPS, LPSA, LPSB). B) Scree Plot Showing the Variance Explained by the First Six Principal Components**.** B). C) Histogram of contribution of the six *B. pseudomallei a*ntigens to principal component 1. Red dashed line represents the average contribution of antigens. D) Histogram of contribution of the six *B. pseudomallei a*ntigens to principal component 2. Red dashed line represents the average contribution of antigens.

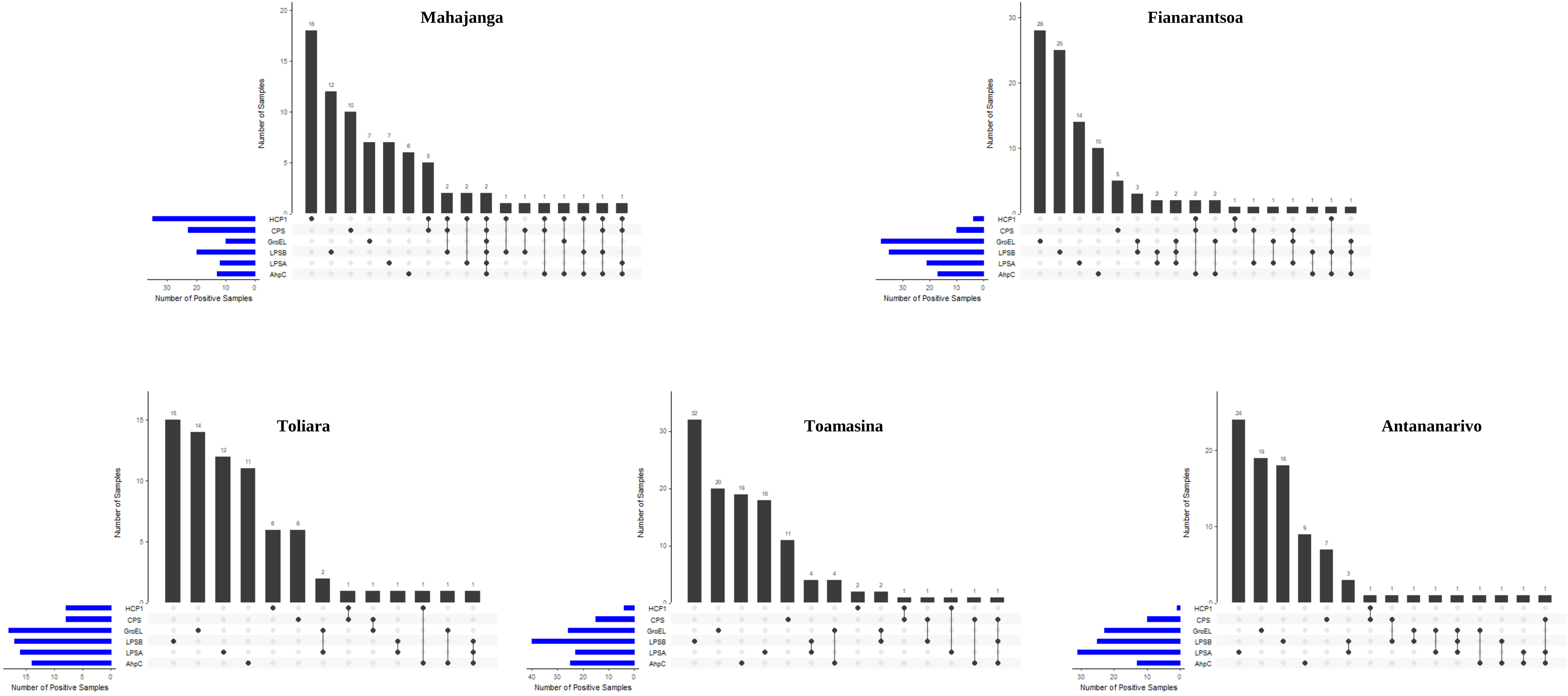

Fig C : UpSet plot depicting the intersection of seropositivity across the six *Burkholderia pseudomallei* antigens. The horizontal bars represent the total number of samples reactive to each individual antigen, while the vertical bars indicate the number of samples exhibiting reactivity to specific combinations of antigens. Dots connected by lines in the matrix below the vertical bars denote antigen combinations.
